# Supplementary material for: Association between depressive symptoms and objectively measured daily step count in individuals at high risk of cardiovascular disease in South London, UK: a cross-sectional study
Source: BMJ Open. 2018 Apr 12;8(4):e020942. doi: 10.1136/bmjopen-2017-020942 (PMC5898324; doi:10.1136/bmjopen-2017-020942)
Supplement: Supplementary data [file bmjopen-2017-020942supp001.pdf]

Supplementary Table 1: Estimated effects (coefficients and relative % change) of individual characteristics on average daily steps in subjects at high risk of CVD with ≥5 valid wear days

Variables		Model 1[a]		Model 2[b]	
		Coefficient	Relative % Change (95% CI)	Coefficient	Relative % Change (95% CI)
**Constant**		8.834		8.942	
**Basic confounders**					
**Age** (impact of additional year)		0.002	0.2 (-0.3 to 0.7)	-0.013***	-1.3 (-1.8 to -0.7)
**Gender**	Male	0	0	0	0
	Female	-0.239***	-21.3 (-25.9 to -16.4)	-0.143***	-13.4 (-18.4 to -7.9)
**Day**	Weekday	0	0	0	0
	Weekend	-0.162***	-14.9 (-16.6 to -13.2)	-0.161***	-14.9 (-16.6 to -13.2)
**Season**	Spring	0	0	0	0
	Summer	-0.001	-0.1 (-6.1 to 6.3)	0.011	1.1 (-4.8 to 7.3)
	Autumn	-0.005	-0.5 (-6.4 to 5.8)	-0.005	-0.5 (-6.2 to 5.6)
	Winter	-0.095**	-9.0 (-14.4 to -3.3)	-0.102**	-9.7 (-14.9 to -4.2)
**a) Depressive Symptoms constant**		8.854			
None (PHQ-9: 0 - 4)		0	0	0	0
Mild (PHQ-9: 5 - 9)		-0.149***	-13.9 (-19.4 to -7.9)	-0.124***	-11.7 (-17.2 to -5.8)
Moderate/Severe (PHQ-9≥10)		-0.171**	-15.7 (-24.7 to -5.6)	-0.135**	-12.6 (-21.5 to -2.7)
**b) Ethnicity constant**		8.848			
White		0	0	0	0
Black/Asian/Other		-0.136***	-12.7 (-18.5 to -6.4)	-0.193***	-17.5 (-23.1 to -11.5)
**c) Education level constant**		8.872			
Level 3: A Level or higher		0	0	0	0
Level 2: O Level/GCSE/NVQ		-0.072**	-7.0 (-11.5 to -2.1)	-0.058*	-5.6 (-10.1 to -1.0)
Level 1: no formal qualification		-0.087**	-8.3 (-13.0 to -3.4)	-0.075**	-7.2 (-11.8 to -2.5)
**d) BMI constant**		8.830			
Impact of one unit BMI increase		-0.025***	-2.5 (-3.0 to -2.0)	-0.028***	-2.8 (-3.3 to -2.3)
**e) Smoking status constant**		8.863			
Never smoked		0	0	0	0
Ex-smoker		-0.016	-1.5 (-6.1 to 3.3)	-0.027	-2.7 (-7.1 to 2.0)
Current smoker		-0.120**	-11.3 (-17.2 to -5.0)	-0.189***	-17.2 (-22.6 to -11.4)
**f) AUDIT score constant**		8.844			
Low risk (score 1-7)		0	0	0	0
Possibly harmful (score ≥8)		0.004	0.4 (-5.3 to - 6.5)	0.035	3.6 (-2.1 to 9.7)
Abstainer (score 0)		-0.119**	-11.3 (-17.3 to -4.8)	-0.089*	-8.6 (-14.7 to -1.9)

[a]*Model 1: all variables adjusted for basic confounders (age, gender, day, season). Note: There is a constant for the basic confounders only model and separate constants for this model adjusted for each additional confounder (a-f).* [b]*Model 2: all variables mutually adjusted, thus there is only one constant for the whole model. Reference group within each category is set to 0. AUDIT:* Alcohol Use Disorders Identification Test. *Sign. levels: *<.05, **<.01, ***<.001.*
