# Supplementary material for: Association between depressive symptoms and objectively measured daily step count in individuals at high risk of cardiovascular disease in South London, UK: a cross-sectional study
Source: BMJ Open. 2018 Apr 12;8(4):e020942. doi: 10.1136/bmjopen-2017-020942 (PMC5898324; doi:10.1136/bmjopen-2017-020942)
Supplement: Supplementary data [file bmjopen-2017-020942supp002.pdf]

Supplementary Table 2: Estimated effects (coefficients and relative % change) of individual characteristics on average daily steps in subjects at high risk of CVD with a QRisk2 ≥ 20%

Variables		Model 1[a]		Model 2[b]	
		Coefficient	Relative % Change (95% CI)	Coefficient	Relative % Change (95% CI)
**Constant**		8.803		8.918	
**Basic confounders**					
**Age** (impact additional year)		0.004***	0.4 (-0.1 to 1.0)	-0.011***	-1.1 (-1.7 to -0.5)
**Gender**	Male	0	0	0	0
	Female	-0.225***	-20.1 (-25.2 to -14.7)	-0.133***	-12.4 (-18.0 to -6.5)
**Day**	Weekday	0	0	0	0
	Weekend	-0.162***	-15.0 (-16.7 to -13.2)	-0.162***	-15.0 (-16.7 to -13.1)
**Season**	Spring	0	0	0	0
	Summer	-0.002	-0.3 (-6.4 to 6.4)	0.0128	1.3 (-4.8 to 7.8)
	Autumn	-0.015	-1.5 (-7.6 to 5.0)	-0.018	-1.8 (-7.6 to 4.5)
	Winter	-0.094**	-9.0 (-14.6 to -3.0)	-0.103**	-9.8 (-15.2 to -4.0)
**a) Depressive Symptoms constant**		8.830			
None (PHQ-9: 0 - 4)		0	0	0	0
Mild (PHQ-9: 5 - 9)		-0.177***	-16.2 (-21.7 to -10.4)	-0.148***	-13.8 (-19.3 to -7.9)
Moderate/Severe (PHQ-9≥10)		-0.234***	-20.9 (-29.3 to -11.5)	-0.206***	-18.6 (-26.9 to -9.3)
**b) Ethnicity constant**		8.817			
White		0	0	0	0
Black/Asian/Other		-0.133***	-12.4 (-18.6 to -5.7)	-0.181***	-16.6 (-22.6 to -10.1)
**c) Education level constant**		8.835			
Level 3: A Level or higher		0	0	0	0
Level 2: O Level/GCSE/NVQ		-0.059*	-5.7 (-10.6 to -0.6)	-0.047	-4.6 (-9.4 to -0.4)
Level 1: no formal qualification		-0.073**	-7.0 (-11.9 to -1.9)	-0.056*	-5.5 (-10.3 to -0.4)
**d) BMI constant**		8.804			
BMI per one unit increase		-0.023***	-2.3 (-2.8 to -1.8)	-0.026***	-2.6 (-3.1 to -2.1)
**e) Smoking status constant**		8.835			
Never smoked		0	0	0	0
Ex-smoker		-0.015	-1.5 (-6.4 to 3.7)	-0.028	-2.8 (-7.5 to 2.2)
Current smoker		-0.139***	-12.9 (-19.0 to -6.5)	-0.203***	-18.4 (-24.0 to -12.3)
**f) AUDIT score constant**		8.810			
Low risk (score 1-7)		0	0	0	0
Possibly harmful (score ≥8)		0.011	1.1 (-4.9 to 7.5)	0.042	4.25 (-1.7 to 10.7)
Abstainer (score 0)		-0.095*	-9.1 (-15.6 to -2.1)	-0.072	-7.0 (-13.6 to 0.2)

[a]*Model 1: all variables adjusted for basic confounders (age, gender, day, season). Note: There is a constant for the basic confounders only model and separate constants for this model adjusted for each additional confounder (a-f).* [b]*Model 2: all variables mutually adjusted, thus there is only one constant for the whole model. Reference group within each category is set to 0. AUDIT:* Alcohol Use Disorders Identification Test. *Sign. levels: *<.05, **<.01, ***<.001.*
