# Supplementary material for: Association between depressive symptoms and objectively measured daily step count in individuals at high risk of cardiovascular disease in South London, UK: a cross-sectional study
Source: BMJ Open. 2018 Apr 12;8(4):e020942. doi: 10.1136/bmjopen-2017-020942 (PMC5898324; doi:10.1136/bmjopen-2017-020942)
Supplement: Author's manuscript [file bmjopen-2017-020942.draft_revisions.pdf]

BMJ Open

BMJ Open is committed to open peer review. As part of this commitment we make the peer review history of every article we publish publicly available.

When an article is published we post the peer reviewers' comments and the authors' responses online. We also post the versions of the paper that were used during peer review. These are the versions that the peer review comments apply to.

The versions of the paper that follow are the versions that were submitted during the peer review process. They are not the versions of record or the final published versions. They should not be cited or distributed as the published version of this manuscript.

BMJ Open is an open access journal and the full, final, typeset and author-corrected version of record of the manuscript is available on our site with no access controls, subscription charges or pay-per-view fees (http://bmjopen.bmj.com).

If you have any questions on BMJ Open's open peer review process please email
info.bmjopen@bmj.com

BMJ Open

The association between depressive symptoms and objectively measured daily step count in individuals at high risk of cardiovascular disease: a cross-sectional study

Journal:	*BMJ Open*
Manuscript ID	bmjopen-2017-020942
Article Type:	Research
Date Submitted by the Author:	04-Dec-2017
Complete List of Authors:	Ludwig, Vera; Technische Universitat Dresden Medizinische Fakultat Carl Gustav Carus, Department of Medicine III<br>Bayley, Adam; Institute of Psychiatry, Psychology and Neuroscience, King's College London, Department of Psychological Medicine<br>Cook, Derek; St George's University, Population Health Research Institute<br>Stahl, Daniel; Institute of Psychiatry, Psychology and Neuroscience, King's College London, Department of Biostatistics and Health Informatics<br>Treasure, Janet; Institute of Psychiatry, Psychology and Neuroscience, King's College London, Section of Eating Disorders<br>Asthworth, Mark; King's College London, UK, Primary Care and Public Health Sciences<br>Greenough, Anne; Kings College London, Department of Women and Children's Health, School of Life Sciences, Faculty of Life Science and Medicine; King's College London, 7MRC & Asthma UK Centre in Allergic Mechanisms of Asthma<br>Winkley, Kirsty; Institute of Psychiatry, Psychology and Neuroscience, King's College London, Department of Psychological Medicine<br>Bornstein, Stefan ; University Hospital Carl Gustav Carus, Technical University Dresden, Department of Medicine III<br>Ismail, Khalida; Institute of Psychiatry, Psychology and Neuroscience, King's College London, Department of Psychological Medicine
Keywords:	Adult psychiatry < PSYCHIATRY, PUBLIC HEALTH, SPORTS MEDICINE

SCHOLARONE™
Manuscripts

The association between depressive symptoms and objectively measured daily step count in individuals at high risk of cardiovascular disease: a cross-sectional study

Vera M. Ludwig[1], Adam Bayley[2], Derek G. Cook[3], Daniel Stahl[4], Janet L. Treasure[5], Mark Ashworth[6], Anne Greenough[7-9], Kirsty Winkley[2], Stefan R. Bornstein[1], Khalida Ismail[2]

[1]Department of Medicine III, University Hospital Carl Gustav Carus, Technical University Dresden, Germany.

[2]Department of Psychological Medicine, Institute of Psychiatry, Psychology and Neuroscience, King's College London, UK.

[3]Population Health Research Institute, St George's, University of London, UK.

[4]Department of Biostatistics and Health Informatics, Institute of Psychiatry, Psychology and Neuroscience, King's College London, UK.

[5]Section of Eating Disorders, Institute of Psychiatry, Psychology and Neuroscience, King's College London, UK.

[6]Department of Primary Care and Public Health Sciences, King's College London, UK.

[7]MRC & Asthma UK Centre in Allergic Mechanisms of Asthma, King's College London.

[8]Department of Women and Children's Health, School of Life Course Sciences, Faculty of Life Sciences and Medicine, Kings College London.

[9]NIHR Biomedical Research Centre at Guy's and St Thomas' NHS Foundation Trust and King's College London.

Corresponding author: Khalida Ismail, Department of Psychological Medicine, King's College London, 10 Cutcombe Road, London SE5 9RJ, UK. Tel: +44 (0)20 7848 5131. E-mail: khalida.2.ismail@kcl.ac.uk

Word count: 3,491 words

Key words: physical activity, accelerometer, PHQ-9, QRisk2, CVD

For peer review only

ABSTRACT

Objectives: Depressive symptoms are common, but rarely considered as a risk factor for unhealthy lifestyles associated with cardiovascular disease (CVD). This study investigates whether depressive symptoms are associated with reduced physical activity (PA) in individuals at high risk of developing CVD.

Design: Secondary analysis of the cross-sectional baseline data from an intensive lifestyle intervention trial (MOVE-IT).

Setting: 135 primary care practises in south London, UK.

Participants: 1,742 adults, 49-74 years, 86% male at high (≥20%) risk of developing CVD in the next 10 years (as defined via QRisk2-Score).

Outcome measures: The main explanatory variable was depressive symptoms measured via the Patient Health Questionnaire-9 (PHQ-9). The main outcome was daily step count measured with an accelerometer (ActiGraph GT3X) stratified by weekdays and weekend days.

Results: The median daily step count of the total sample was 6,151 (IQR, 3,510) with significant differences ($p<.001$) in mean daily step count between participants with low (PHQ-9 score 0 to 4), mild (PHQ-9 score 5 to 9), and moderate to severe depressive symptoms (PHQ-9 score ≥10). Controlling for age, gender, ethnicity, education level, body mass index (BMI), smoking, consumption of alcohol, day of the week, and season, individuals with mild depressive symptoms, and those with moderate to severe depressive symptoms, walked 13.5% (CI 18.8 to 7.9) and 15.6% (CI 23.7 to 6.5) less than non-depressed individuals respectively. Furthermore, male gender, white ethnicity, higher education level, lower BMI, non-smokers, moderate alcohol intake, weekdays, and summer season were independently associated with higher step count.

Conclusions: Depressive symptoms are associated with lower step count in people at high risk of CVD. This may potentially modify the effectiveness of lifestyle interventions to reduce the risk of CVD.

Trial registration: ISRCTN84864870

For peer review only - http://bmjopen.bmj.com/site/about/guidelines.xhtml

ARTICLE SUMMARY

Strengths and limitations of this study

- This study is the first to look at the relationship between depressive symptoms, and objectively measured physical activity in a large sample (n=1,720) of individuals at high risk of developing CVD.
- The analysis controls for potential confounding of: age, gender, ethnicity, education level, BMI, smoking, consumption of alcohol, day of the week, and season.
- The inclusion of participants with as little as one valid wear day tries to minimise bias by not excluding more depressed participants that did not wear the monitor the requested five days.
- No causalities can be inferred from the cross-sectional data.
- As it is a sample from the general population, the proportion of severely depressed subjects is small, and the power to compare the severity categories of depressive symptoms on step count is limited.

INTRODUCTION

Cardiovascular disease (CVD) remains the most common cause of death worldwide: it accounts for a third of global mortality,[1] and contributes considerably to years lost to disability.[2] The main lifestyle risk factors for CVD are tobacco smoking, physical inactivity, unhealthy diets, and the harmful use of alcohol.[1] Estimations suggest that increasing physical activity (PA) to the World Health Organisation recommended amount would reduce the global CVD related burden by 6%.[3] It is increasingly recognised that depression (defined here as an umbrella term for depressive disorder and depressive symptoms) is an independent risk factor for CVD,[4] and is associated with increased risk of mortality after a CVD event,[5] but mental health is not routinely considered as a factor that may affect uptake of PA to improve CVD outcomes.

only

For most individuals, and especially the elderly, the most accessible and safest form of PA is walking. Increased number of daily steps, independent of walking speed or intensity, is associated with improved biomedical outcomes such as lower body mass index (BMI),[6] lower cholesterol ratio and triglyceride levels,[7] and a lower rate of CVD incidents overall.[8] Self-report questionnaires to assess walking are prone to over and under reporting biases.[9] Objectively measured walking such as accelerometers or pedometers can overcome this limitation.[10] Potential confounders of any association between depressive symptoms and walking include younger age, male gender, lower BMI and white ethnicity, which are associated with higher step count.[11–14] Education is also a potential confounder although there have been mixed findings.[11,12,15] There are also well known time effects namely that people walk more on weekdays than on weekends and during summer months than in winter.[14]

The relationship between depressive symptoms and step count have only been assessed in specific populations with small sample sizes, such as low-socioeconomic status Latino immigrants,[16] elderly Japanese people,[17] patients with chronic conditions such as heart failure,[18,19] or chronic obstructive pulmonary disease.[20,21] These studies yield contradictory results, with some observing no association between depressive symptoms and daily step count,[19,21] while others report a negative correlation.[16–18,20] In one cross sectional sample of healthy older adults, an inverse association between depressive symptoms and accelerometer measured daily step count, using the Goldberg Depression Scale-15, disappeared after controlling for general health and disability.[22] While a systematic review suggests reduced levels of objectively measured PA in depressed patients,[23] it is not known whether this association is present in those at high risk of CVD and taken into account important confounding such as gender and age.

We aim to describe the distribution of objectively measured average daily step count in a sample at high risk of CVD, and to test the hypothesis that individuals with greater

For peer review only

depressive symptoms are likely to have reduced daily step counts, controlling for potential confounding.

METHODS AND MATERIAL

Design

This is a cross-sectional analysis of the baseline data of individuals at high risk of CVD who were participating in a randomised controlled trial (RCT) testing the effectiveness of an intensive lifestyle intervention 'MOtiVational intErviewing InTervention' (MOVE IT) to increase PA and reduce weight. The data was collected before participants were randomised.

Setting and sampling frame

The MOVE IT study was conducted in the 12 south London Clinical Commissioning Groups (CCGs), which are the local administrative authorities for commissioning health care for its residents, representing a diverse socioeconomic and ethnic population of approximately three million UK residents. All primary care practices with a list size of >5,000 registered patients, resident within these CCGs were invited to participate. The patient inclusion criteria were: ≥20% risk of having a fatal or non-fatal CVD event in the next 10 years as defined by the QRisk2 score (2013 version);[24] age ≥40 and ≤74 years; permanent residency, and fluent in English. The exclusion criteria were: known CVD; on the general practice electronic register for diabetes, chronic kidney disease, atrial fibrillation or stroke; chronic obstructive pulmonary disease or morbid obesity (BMI>50kg/m^2). Participants were recruited from June 7th 2013 to February 19th 2015. Ethical approval was granted by the Dulwich Ethics Committee (reference: 12/LO/0917), and all participants gave written informed consent. Further details of the trial protocol are available in Bayley et al.[25]

Measures

The main explanatory variable was self-report depressive symptoms using the Patient Health Questionnaire-9 (PHQ-9).[26] The PHQ-9 scores the occurrence of each of the nine

For peer review only

Diagnostic and Statistical Manual of Mental Disorders 4th Edition (DSM-IV) criteria for major depressive disorder over the past two weeks on a scale from 0 (not at all) to 3 (nearly every day). The total sum score, ranging from 0 to 27, can be categorised as not depressed (0–4), mild (5–9), moderate (10–14), moderately severe (15–19), and severe depression (20–27).[26] A score of ten or above defined as clinically relevant depression, and a recent meta-analysis reported that a cut-off score ≥10 generated a sensitivity of 77% and specificity of 85% in diagnosis for major depression.[27] In our study the number of individuals with scores in the three top categories (10–14, 15–19 and 20–27) was low (2.9, 0.9, and 0.2% respectively), therefore we merged them to create one category of moderate to severe 'clinical' depression (score ≥10).

The main outcome was the average daily number of steps measured with the ActiGraph GT3X accelerometer (ActiGraph, Florida, United States), a tri-axial movement sensor that has been validated on number of steps taken and is reported to record 98.5% of steps.[28] Number of steps correlated (r=.70) with average moderate-to-vigorous physical activity (MVPA). We chose steps/day as the main outcome measure in this analysis as it was specified as the main outcome measure in the MOVE-IT trial and walking is the main form of physical activity in this elderly population. We also note that steps per day was highly correlated with minutes of MVPA (r=.75). Steps also has the merit that is an easily understood measure which facilitates clinical application of the results. The accelerometers were given to the participants by a researcher who advised them to attach the monitor to the waist and wear it for seven days from waking in the morning until going to bed at night, only removing it for bathing and swimming. If monitors were worn for less than nine hours (540 minutes) per day, these wear days were considered missing as it suggests the participant forgot to wear the monitor for a part of the day. The aim was to generate a minimum of five valid wear days per participant to ensure the collected data represents habitual activity levels.[29] A preliminary analysis revealed that people who wore the monitor for less than five days exhibited significantly more depressive symptoms that those who wore it for at least

For peer review only - http://bmjopen.bmj.com/site/about/guidelines.xhtml

five days so to reduce bias we included individuals who had less than five days of wear time. We therefore included all participants with at least one valid wear day in the analysis and performed a sensitivity analysis to determine whether including individuals with one wear day only altered the results.

To account for potential confounding, reported covariates of depressive symptoms and objectively measured PA (age, gender, BMI, ethnicity, day of the week, level of education,[11] season,[14] smoking, and alcohol intake[30]) were controlled for in the analysis. Age, gender and ethnicity were assessed using standardised questions. Educational levels were created categorising participants into level 1: no formal qualifications; level 2: qualifications at the end of compulsory school education in UK typically at age 16 years; or level 3: qualifications awarded at the end of further education typically at age 18 years. Lifestyle covariates were: self-report smoking status, which was categorised as never smoked, ex-smoker and current smoker and the Alcohol Use Disorders Identification Test (AUDIT), which is a 10-item questionnaire that categorised alcohol consumption as abstainers (AUDIT score= 0), low risk (score 1-7), and possibly hazardous or harmful drinkers (score ≥8).[31]

Statistical Analysis

Data on daily step count were downloaded from the activity monitors using Actilife Version 6.11.7.[32] The number of valid wear days (≥540 minutes) per participant was counted. Extreme outliers in daily step count were individually investigated to ensure plausibility and rule out device failure. This was not the case and no cases were removed from the analysis. Measurements of step count for weekdays (Monday to Friday) and weekend days (Saturday, Sunday) were merged to create one mean step count for each of the two categories, thereby creating at least one and at most two measurements per participant. Baseline characteristics were summarised as means (± standard deviation) or percentages. As the step count data was not normally distributed, median and its interquartile range (IQR) are presented, and

For peer review only

non-parametric Spearmen rank correlation were applied. Independent sample t-tests were employed to compare depression scores between subsamples (≥5 vs. <5 wear days, ≥20% and <20% QRisk2).

For the main analysis, a linear mixed model (LMM) was used. LMMs can account for the dependency of repeated observations of the same subject (weekdays and weekends) and allow including all individuals with at least one outcome observation.[33] To improve the fit of the model, the step count data were log-transformed before they were entered into the model. In order to investigate the sizes of the adjusted and unadjusted effects of each predictor on step count, we introduced two models. The first model aims to describe the individual associations between each predictor and mean daily step count. We controlled for age, gender, season and day of accelerometer wear (basic confounders) and then individually entered each additional covariate, a) depressive symptoms, b) ethnicity, c) education level, d) BMI, e) smoking status and f) drinking of alcohol, into the LMM as independent predictor for log step count, obtaining the estimated change caused by each of the predictors. In the second model, all variables were entered simultaneously to determine the mutually adjusted effects of all predictors. Age and BMI were entered as continuous fixed effects variables (centred around the mean) and the others were entered as categorical fixed effects variables. We used an unstructured covariance structure for the LMM. Assumptions of the LMM were assessed by visual inspection of the residual plots.[34] The constant for each LMM is reported alongside the coefficients of relative change for each variable. These relative differences between the log transformed step counts were back transformed to the percentage difference in step count by applying the formula $100(e^{\text{Coefficient}}-1)$.

A sensitivity analysis was conducted to determine if wearing the accelerometer for less than five days alters the results by rerunning the main analyses on participants with at least five wear days (Supplementary Table 1). Reanalysis of QRisk2 scores using cleaned data and the QRisk2 2013 batch calculator revealed that 155 (9%) of the included participants had a

For peer review only - http://bmjopen.bmj.com/site/about/guidelines.xhtml

QRisk2 score <20%. We conducted a second sensitivity analysis by rerunning the main analyses including only patients with a QRisk2 score of ≥20 (Supplementary Table 2) to check for potential bias. Data were analysed using IBM SPSS Statistics for Macintosh, Version 24.0 (SPSS Inc., Chicago, IL) was used for all analyses, p-values <.05 were considered significant.

RESULTS

Population Characteristics

The MOVE IT sample consisted of 135 primary care practices in the 12 south London CCGs, from which 1,742 participants were eligible on screening, and consented to participate. At baseline, only n=22 (1.3%) provided less than one valid wear day with ≥540 minutes and had to be excluded right away. Additionally, 117 participants (6.7%) provided at least one, but less than the requested five full wear days. When comparing these 117 participants with those who wore the monitor for five days or more, we found that the former were significantly more depressed than the latter ($p<.001$). If we excluded this group, this may lead to an underestimation of the effect of depression on step count therefore we decided to include this group, giving a final sample of 1,720. The average QRisk2 score of the sample was 25% (SD 4.9). On re-calculation of QRisk2 scores, 155 patients with a score <20% had been recruited into the trial. As there was no difference in depressive symptoms compared to those with QRisk2 score ≥20% ($p=.394$), we retained this subgroup in the analysis. The mean age was 69 (SD 4.1) years, the vast majority were male, and of white ethnicity. Demographic and behavioural characteristics of the final sample stratified by accelerometer wear day are reported in Table 1.

Step count data

A total of 11,140 wear days from n=1,720 participants were included in the analysis. These participants provided a mean of 6.5 (SD 1.2) wear days. Daily step count ranged from 105 to 21,313 steps with a median of 6,151 (IQR 3,510). Figure 1 shows an inverse relationship

For peer review only - http://bmjopen.bmj.com/site/about/guidelines.xhtml

(Spearman's rho r=-.149, p=.01) between depressive symptom score and mean individual daily step count. Step count is also associated with gender, ethnicity, education level, BMI, smoking, drinking of alcohol, and with weekdays, but not age (Table 1).

For peer review only

Table 1 Demographic characteristics and distribution of median daily step count in a randomised controlled trial assessing the effectiveness of an intensive lifestyle intervention

Variables		Weekdays N (%)	Weekdays Median Steps (IQR)[a]	Weekdays p-value[b]	Weekends N (%)	Weekends Median Steps (IQR)[a]	Weekends p-value[b]
**Total study population**		*1,719 (100)*	*6,447 (3,875)*		*1,645 (100)*	*5,676 (3,737)*	
**Age**	>70	*58 (3.4)*	*6,434 (4,175)*	*p=.859*	*52 (3.2)*	*5,639 (4,209)*	*p=.454*
	50-59[c]	*701 (40.8)*	*6,319 (4,017)*		*661 (40.2)*	*5,784 (3,985)*	
	60-69	*960 (55.8)*	*6,485 (3,738)*		*932 (56.7)*	*5,640 (3,659)*	
**Gender**	Female	*1,470 (85.5)*	*6,665 (3,910)*	*p<.001*	*1,402 (85.2)*	*5,903 (3,870)*	*p<.001*
	Male	*249 (14.5)*	*5,441 (3,343)*		*243 (14.8)*	*4,590 (3,143)*	
**Ethnicity**	White	*1,538 (89.5)*	*6,536 (3,895)*	*p<.001*	*1,472 (89.5)*	*5,800 (3,757)*	*p<.001*
	Black/Asian/Other	*181 (10.5)*	*5,619 (3,493)*		*173 (10.5)*	*4,676 (3,321)*	
**Education level[d]**	Level1	*427 (25.3)*	*6,022 (3,742)*	*p=.001*	*399 (24.7)*	*5,287 (3,713)*	*p=.018*
	Level 2	*467 (27.6)*	*6,391 (4,070)*		*444 (27.5)*	*5,517 (3,907)*	
	Level 3	*796 (47.1)*	*6,734 (3,871)*		*774 (47.9)*	*5,928 (3,690)*	
**Depression (PHQ-9)**	None (0-4)	*1,449 (84.3)*	*6,656 (3,855)*	*p<.001*	*1,394 (84.7)*	*5,804 (3,831)*	*p<.001*
	Mild (5-9)	*201 (11.7)*	*5,563 (3,330)*		*189 (11.5)*	*5,045 (3,473)*	
	Clinical (≥10)	*69 (4.0)*	*5,428 (3,905)*		*62 (3.8)*	*5,258 (3,939)*	
**BMI (kg/m^2)**	<20	*24 (1.4)*	*6,899 (4,774)*	*p<.001*	*22 (1.3)*	*6,178 (3,899)*	*p<.001*
	20-25	*371 (21.6)*	*7,255 (3,983)*		*365 (22.2)*	*6,307 (4,329)*	
	25-30	*800 (46.5)*	*6,550 (3,736)*		*773 (47.0)*	*5,796 (3,615)*	
	>30	*524 (30.5)*	*5,850 (3,594)*		*485 (29.5)*	*5,108 (3,559)*	
**Smoking**	Never smoked	*482 (28.0)*	*6,485 (3,694)*	*p<.001*	*468 (28.4)*	*5,795 (3,760)*	*p=.047*
	Ex-Smoker	*975 (56.7)*	*6,605 (3,850)*		*929 (56.5)*	*5,784 (3,670)*	
	Current Smoker	*262 (15.2)*	*5,795 (4,285)*		*248 (15.1)*	*5,268 (4,115)*	
**Alcohol (AUDIT)**	Abstainer (0)	*176 (10.2)*	*5,533 (4,208)*	*p<.001*	*171 (10.4)*	*4,984 (3,598)*	*p=.001*
	Low risk (1-7)	*1,274 (74.2)*	*6,544 (3,628)*		*1,216 (73.9)*	*5,767 (3,629)*	
	Harmful (≥8)	*269 (15.6)*	*6,490 (4,370)*		*258 (15.7)*	*5,787 (4,027)*	

[a]Median step count and IQR are rounded to the closest full number. [b]p-values are calculated with Mann-Whitney U tests and Kruskall Wallis tests. [c]One person was younger than 50 years (49), this person was included in the ≥50 category. [d]N=29 missing for weekdays, n=28 missing for weekends.

Linear mixed model

The LMMs (Table 2) observed significant associations between depressive symptoms and step count. Controlling for age, gender day and season (Model 1), individuals with moderate to severe depressive symptoms walked 19.0% less (p<.001) than non-depressed individuals, and mildly depressed individuals walked 16.2% less (p<.001). Mutually controlling for all predictors (Model 2) decreased the effect of depressive symptoms to 15.6% and 13.5% respectively (p=.001, p<.001). All other covariates significantly influenced PA as well. In model 2, each additional year in age and one point increase in BMI was associated with a decrease in step count by 1.2% and 2.7% respectively (p<.001, p<.001). Female gender reduced step count by 13.8% (p<.001), individuals from ethnic minorities walked 17.8% less steps (p<.001). Level 3 qualifications were associated with higher step count difference of 5.7% compared to no formal qualifications (p=.022). Current smokers' step count was 19.1% lower compared to people who had never smoked (p<.001) and participants abstaining from alcohol consumption had an 8.8% lower step count than people who are low risk drinkers (p=.010). On weekends 14.8% fewer steps were recorded than on weekdays (p<.001) and during the winter months there was a decrease of 9.3% compared to spring (p=.001). Sensitivity analyses revealed that rerunning the models with participants with at least 5 days of complete wear time (Supplementary Table 1) and participants with QRisk2 score >20 only (Supplementary Table 2), provided similar parameter estimates and did not alter the conclusions of this study.

For peer review only

Table 2 Estimated effects (coefficients and relative % change) of individual characteristics on average daily steps in subjects at high risk of CVD

Variables	Model 1[a] Coefficient	Model 1[a] Relative % Change (95% CI)	Model 2[b] Coefficient	Model 2[b] Relative % Change (95% CI)
**Basic confounders constant**	8.813		8.937	
**Age** (impact additional year)	0.004	0.4 (-0.1 to 1.0)	-0.012***	-1.2 (-1.7 to -0.6)
**Gender** Male	0[c]	0[c]	0[c]	0[c]
Female	-0.249***	-22.1 (-26.7 to -17.2)	-0.149***	-13.8 (-18.9 to -8.5)
**Day** Weekday	0[c]	0[c]	0[c]	0[c]
Weekend	-0.161***	-14.9 (-16.6 to -13.2)	-0.161***	-14.9 (-16.6 to -13.1)
**Season** Spring	0[c]	0[c]	0[c]	0[c]
Summer	-0.007	-0.7 (-6.7 to 5.7)	0.004	0.4 (-5.4 to 6.7)
Autumn	-0.013	-1.3 (-7.2 to 5.0)	-0.015	-1.5 (-7.2 to 4.5)
Winter	-0.086**	-8.3 (-13.8 to -2.4)	-0.098**	-9.3 (-14.5 to -3.8)
**a) Depressive Symptoms constant**	8.840		8.937	
None (PHQ-9: 0 - 4)	0[c]	0[c]	0[c]	0[c]
Mild (PHQ-9: 5 - 9)	-0.177***	-16.2 (-21.5 to -10.6)	-0.142***	-13.3 (-18.6 to -7.6)
Moderate/Severe (PHQ-9≥10)	-0.211***	-19.0 (-27.2 to -10.0)	-0.170**	-15.6 (-23.8 to -6.6)
**b) Ethnicity constant**	8.845		8.937	
White	0[c]	0[c]	0[c]	0[c]
Black/Asian/Other	-0.145***	-13.5 (-19.2 to -7.4)	-0.196***	-17.8 (-23.3 to -12.0)
**c) Education level constant**	8.845		8.937	
Level 3: A Level or higher	0[c]	0[c]	0[c]	0[c]
Level 2: O Level/GCSE/NVQ	-0.063*	-6.1 (-10.7 to -1.2)	-0.054*	-5.3 (-9.7 to -0.6)
Level 1: no formal qualification	-0.072**	-7.0 (-11.7 to -2.0)	-0.196*	-5.7 (-10.4 to -0.9)
**d) BMI constant**	8.811		8.937	
Impact of one unit BMI increase	-0.025***	-2.4 (-2.9 to -1.9)	-0.027***	-2.7 (-3.2 to -2.2)
**e) Smoking status constant**	8.849		8.937	
Never smoked	0[c]	0[c]	0[c]	0[c]
Ex-smoker	-0.023	-2.3 (-6.9 to -2.5)	-0.038	-3.7 (-8.2 to 0.9)
Current smoker	-0.141***	-13.2 (-18.9 to -7.0)	-0.212***	-19.1 (-24.4 to -13.4)
**f) Drinking of Alcohol constant**	8.824		8.937	
Low risk (AUDIT: 1-7)	0[c]	0[c]	0[c]	0[c]
Possibly harmful (AUDIT≥8)	-0.001	-0.1 (-5.8 to 6.0)	0.032	3.3 (-2.4 to 9.3)
Abstainer (AUDIT: 0)	-0.124**	-11.7 (-17.6 to -5.2)	-0.092*	-8.8 (-14.9 to -2.2)

*[a]Model 1: all variables adjusted for basic confounders (age, gender, day, season). [b]Model 2: all variables mutually adjusted. [c]Reference group is set to 0. Significance levels: *<.05, **<.01, ***<.001.*

277

DISCUSSION

Main study findings

This study assessed patterns of PA and depressive symptoms in people at high risk of CVD. We found an inverse relationship where worsening depressive symptoms were associated with significant reductions in step count. Female gender, ethnicity minority, lower education level, higher BMI, smoking, alcohol abstainers, weekends and colder season were independently associated with decreased daily step count.

Study strengths and limitations

The strengths of our study are, firstly, the large sample size, which is representative of the ethnic, socioeconomic, urban and semi-rural diversity typical of large cities in the developed nations. Furthermore, PA was objectively assessed based on accelerometer data. Socio-demographic, biomedical and depression data were collected in face-to-face interviews with trained researchers using standardised schedules. Recruitment was conducted year-round so seasonal variations were accounted for.

The limitations were that the study design was cross-sectional, so causal relationships cannot be inferred, and it remains unclear whether people with depressive symptoms are at increased risk of walking less or less active people are at increased risk for depressive symptoms. Although PA was measured objectively using accelerometers, we cannot rule out that the recorded levels of physical activity are subject to bias, as participants might have changed their performance because they knew they were being observed.[35] This would not, however, have biased estimates of the association with depressive symptoms unless depressed individuals respond differently to wearing accelerometers. The percentage of female participants was small (14.5%), reflecting the lower QRisk2 sores in females.[24] However, data on females with high CVD risk is relevant as the prevalence of depression is higher in females and they have worse outcomes following CVD events.[36] The number of

For peer review only - http://bmjopen.bmj.com/site/about/guidelines.xhtml

study participants with severe depressive symptoms was small, which is to be expected in a primary care population. Our analysis showed that depressive symptom scores were significantly higher in individuals who failed to provide the requested five wear days. We tried to minimise any measurement bias by depression by including all participants with as little as one valid wear day. Finally, as in any study with missing outcome data, we cannot exclude potential bias if missing values do depend on unobserved values (not missing at random).[37]

Comparison with other studies

Normative data suggests that between 2,000 and 9,000 daily steps are a typical estimate of usual daily activity levels in healthy older adults (≥50 years).[13] Our sample falls into that range with an average of 6,515 daily steps. The high inter-individual variability in step count, in our case an IQR of 3,510, is also similar to findings in previous studies.[11,12,15] Women seem to be especially likely to have a sedentary lifestyle, particularly on weekends. Ethnicity, level of deprivation and education level all showed significant influence on step count, indicating the important role of cultural beliefs and awareness of the issue. These findings suggest prevention programmes to increase PA should be targeted at specific subgroups of people at high risk of CVD as well as the general population. The prevalence of clinically relevant depressive symptoms (PHQ-9 score ≥10) in our sample was 4% which is midway between the reported UK prevalence of 2.8% (2.6% in males, 3.0% in females) in the general population [36] and 7% in CVD patients.[38] Our participants did not have CVD and were not on clinical primary care registers for other long-term conditions. However, they were at high risk of developing CVD and the prevalence rate of depressive symptoms were higher than in the general population. This is consistent with the results of a recent Danish register study which reported that depression might be an antecedent for CVD events and mortality therefore another indicator of its importance as an easily measured risk factor.[39]

For peer review only

Previous studies on the association between objectively measured PA and depression yielded inconclusive results. They have reported either a lack of a relationship,[19,21] an inverse association,[16–18,20] or an inverse association that disappeared when controlled for confounding.[22] The studies that reported negative associations between daily step count and depressive symptoms have been conducted in relatively small (n=96 to n=207) [17–19,22], or in highly selective samples such as severe COPD.[20] Our study is the first to examine this relationship in a large sample of community-dwelling older European adults at high risk of CVD who were not known to have a chronic disease.

Implications for clinicians

Practitioners and researchers should consider assessing depressive symptoms when assessing patients' CVD risk or their barriers to uptake of lifestyle interventions. Even mild symptoms of depression (PHQ-9 scores 5 to 9) significantly reduced average daily step count by 13.3%. Similarly, it has been shown that mild symptoms of depression increase mortality risk after acute myocardial infarction.[40] A Cochrane systematic review found that PA interventions were associated with a moderate effect in reducing depressive symptoms compared to no care, and was no more effective than psychological or pharmacological therapies, though these conclusion were based on a few methodologically robust trials.[41] To substantiate this a recent well designed cluster RCT observed that the addition of PA to usual care did not improve depressive symptoms or reduce use of antidepressants compared to usual care.[42] The literature and our results suggest that depressive symptoms may be barrier to increasing PA and that clinicians should consider optimising conventional depression management in order to then improve uptake of PA, which in turn could reduce CVD risk.[3,4]

Implications for future work

The varying patterns of PA in individuals at high risk of CVD indicate that it might be most efficient to target PA interventions specifically towards those with lowest activity ratings, e.g.

For peer review only

females, those from African, Caribbean, or Asian ethnicities, those less educated, or less affluent. Additionally, our results suggest that there is a moderate association between depressive symptoms and daily step count in individuals at high risk of CVD, and that this should be considered as a potential modifying factor in clinical trials for lifestyle interventions in CVD research.

For peer review only

ACKNOWLEDGEMENTS

We would like to thank the research team, healthy lifestyle facilitators, patient volunteers and the South London Clinical Research Network, participating general practices and their patients. We would also like to thank the Trial Steering Committee: Professor Steve Iliffe (Chair), University College London; Professor Tom Marshall, University of Birmingham; Professor James Carpenter, London School of Hygiene and Tropical Medicine, MRC Clinical Trials Unit; Dr Tim Anstiss, independent medical doctor and consultant. We would also like to thank the Data Ethics and Monitoring Committee: Professor Betty Kirkwood (previous Chair), London School of Hygiene and Tropical Medicine and Professor Helen Weiss (Chair), London School of Hygiene and Tropical Medicine; Professor Stephanie Taylor, Queen Mary University of London, Barts and The London School of Medicine and Dentistry, Blizard Institute; Dr David Blane, Imperial College London.

COMPETING INTERESTS STATEMENT

KI discloses that she has received honorarium from Eli-Lilly, Sanofi, Janssen and Sunovion for lectures at educational events. However, she states that this does not constitute a conflict of interest for the present study. The other authors declare no conflict of interest.

FUNDING

The MOVE IT study is funded by the National Institute for Health Research - Health Technology Assessment Programme (NIHR-HTA) (Project: 10/62/03) and work will be published in the Health Technology Assessment journal. The research attachment of VML to King's College London in London, UK, was funded by the International Network on Diabetes and Depression (IN2D), which is financed by the German Federal Ministry of Education and Research (BMBF). DS received partial funding by the National Institute for Health Research (NIHR) Biomedical Research Centre at South London, and Maudsley NHS Foundation Trust and King's College London.

For peer review only

Author Contributions

KI drafted the initial hypothesis. VML developed the protocol, especially supported by AB who, as trial manager, had knowledge of all study particulars and had helped acquire the data. Further, VML wrote the first draft of the report, and performed the literature search Data analysis was conducted by DS, DGC, and VML. JLT, MA, AG and KW helped interpret the data and gave important feedback in the revision process. SRB contributed to the research funding, KI and SRB coordinated the research and oversaw the project. All authors critically reviewed the manuscript for important intellectual content and approved the final manuscript.

Ethics approval

Ethical approval was granted by the Dulwich Ethics Committee (reference: 12/LO/0917) and all participants gave written informed consent.

Data sharing statement

The full data set and statistical code are available from the corresponding author Prof. Khalida Ismail at khalida.2.ismail@kcl.ac.uk upon reasonable request.

For peer review only

REFERENCES

1 Mendis S, Puska P, Norrving B, *et al.*, editors. *Global atlas on cardiovascular disease prevention and control*. Geneva: : World Health Organization in collaboration with the World Heart Federation and the World Stroke Organization 2011.

2 World Health Organization. Global Health Estimates 2015: Disease burden by Cause, Age, Sex, by Country and by Region, 2000-2015. 2016.http://www.who.int/healthinfo/global_burden_disease/estimates/en/index2.html (accessed 14 May 2017).

3 Lee I-M, Shiroma EJ, Lobelo F, *et al.* Effect of physical inactivity on major non-communicable diseases worldwide: an analysis of burden of disease and life expectancy. *The Lancet* 2012;**380**:219–29. doi:10.1016/S0140-6736(12)61031-9

4 Carney RM, Freedland KE. Depression and coronary heart disease. *Nat Rev Cardiol* 2016;**14**:145–55. doi:10.1038/nrcardio.2016.181

5 Hare DL, Toukhsati SR, Johansson P, *et al.* Depression and cardiovascular disease: a clinical review. *Eur Heart J* 2014;**35**:1365–72. doi:10.1093/eurheartj/eht462

6 Tudor-Locke C, Ainsworth BE, Whitt MC, *et al.* The relationship between pedometer-determined ambulatory activity and body composition variables. *Int J Obes Relat Metab Disord J Int Assoc Study Obes* 2001;**25**:1571–8. doi:10.1038/sj.ijo.0801783

7 Brown H, Becker F, Antwi K. Association Between Lipid Biomarkers, Physical Activity, and Socioeconomic Status in a Population-Based Cross-Sectional Study in the UK. *Sports Med - Open* 2016;**2**. doi:10.1186/s40798-016-0049-9

8 Hamer M, Chida Y. Walking and primary prevention: a meta-analysis of prospective cohort studies. *Br J Sports Med* 2008;**42**:238–43. doi:10.1136/bjsm.2007.039974

9 Prince SA, Adamo KB, Hamel M, *et al.* A comparison of direct versus self-report measures for assessing physical activity in adults: a systematic review. *Int J Behav Nutr Phys Act* 2008;**5**:56. doi:10.1186/1479-5868-5-56

10 Taraldsen K, Chastin SFM, Riphagen II, *et al.* Physical activity monitoring by use of accelerometer-based body-worn sensors in older adults: A systematic literature review of current knowledge and applications. *Maturitas* 2012;**71**:13–9. doi:10.1016/j.maturitas.2011.11.003

11 Tudor-Locke C, Ham SA, Macera CA, *et al.* Descriptive epidemiology of pedometer-determined physical activity. *Med Sci Sports Exerc* 2004;**36**:1567–73.

12 Inoue S, Ohya Y, Odagiri Y, *et al.* Sociodemographic Determinants of Pedometer-Determined Physical Activity Among Japanese Adults. *Am J Prev Med* 2011;**40**:566–71. doi:10.1016/j.amepre.2010.12.023

13 Tudor-Locke C, Hart TL, Washington TL. Expected values for pedometer-determined physical activity in older populations. *Int J Behav Nutr Phys Act* 2009;**6**:59. doi:10.1186/1479-5868-6-59

14 Davis MG, Fox KR, Hillsdon M, *et al.* Objectively Measured Physical Activity in a Diverse Sample of Older Urban UK Adults: *Med Sci Sports Exerc* 2011;**43**:647–54. doi:10.1249/MSS.0b013e3181f36196

For peer review only

15 Kari JT, Pehkonen J, Hirvensalo M, *et al.* Income and Physical Activity among Adults: Evidence from Self-Reported and Pedometer-Based Physical Activity Measurements. *PLOS ONE* 2015;**10**:e0135651. doi:10.1371/journal.pone.0135651

16 Drieling RL, Goldman Rosas L, Ma J, *et al.* Community Resource Utilization, Psychosocial Health, and Sociodemographic Factors Associated with Diet and Physical Activity among Low-Income Obese Latino Immigrants. *J Acad Nutr Diet* 2014;**114**:257–65. doi:10.1016/j.jand.2013.07.025

17 Yoshiuchi K, Nakahara R, Kumano H, *et al.* Yearlong Physical Activity and Depressive Symptoms in Older Japanese Adults: Cross-Sectional Data from the Nakanojo Study. *Am J Geriatr Psychiatry* 2006;**14**:621–4. doi:10.1097/01.JGP.0000200602.70504.9c

18 Alosco ML, Spitznagel MB, Miller L, *et al.* Depression is associated with reduced physical activity in persons with heart failure. *Health Psychol* 2012;**31**:754–62. doi:10.1037/a0028711

19 Dontje ML, van der Wal MHL, Stolk RP, *et al.* Daily Physical Activity in Stable Heart Failure Patients: *J Cardiovasc Nurs* 2013;**29**:218–26. doi:10.1097/JCN.0b013e318283ba14

20 Barriga S, Rodrigues F, Bárbara C. Factors that influence physical activity in the daily life of male patients with chronic obstructive pulmonary disease. *Rev Port Pneumol* 2014;**20**:131–7. doi:10.1016/j.rppneu.2013.09.004

21 Di Marco F, Terraneo S, Roggi MA, *et al.* Physical Activity Impairment in Depressed COPD Subjects. *Respir Care* 2014;**59**:726–34. doi:10.4187/respcare.02848

22 Harris TJ, Owen CG, Victor CR, *et al.* What factors are associated with physical activity in older people, assessed objectively by accelerometry? *Br J Sports Med* 2009;**43**:442–50. doi:10.1136/bjsm.2008.048033

23 Burton C, McKinstry B, Szentagotai Tătar A, *et al.* Activity monitoring in patients with depression: A systematic review. *J Affect Disord* 2013;**145**:21–8. doi:10.1016/j.jad.2012.07.001

24 Collins GS, Altman DG. Predicting the 10 year risk of cardiovascular disease in the United Kingdom: independent and external validation of an updated version of QRISK2. *BMJ* 2012;**344**:e4181–e4181. doi:10.1136/bmj.e4181

25 Bayley A, de Zoysa N, Cook DG, *et al.* Comparing the effectiveness of an enhanced MOtiVational intErviewing InTervention (MOVE IT) with usual care for reducing cardiovascular risk in high risk subjects: study protocol for a randomised controlled trial. *Trials* 2015;**16**. doi:10.1186/s13063-015-0593-5

26 Kroenke K, Spitzer RL, Williams JBW. The PHQ-9: Validity of a brief depression severity measure. *J Gen Intern Med* 2001;**16**:606–13. doi:10.1046/j.1525-1497.2001.016009606.x

27 Manea L, Gilbody S, McMillan D. A diagnostic meta-analysis of the Patient Health Questionnaire-9 (PHQ-9) algorithm scoring method as a screen for depression. *Gen Hosp Psychiatry* 2015;**37**:67–75. doi:10.1016/j.genhosppsych.2014.09.009

28 Lee JA, Williams SM, Brown DD, *et al.* Concurrent validation of the Actigraph gt3x+, Polar Active accelerometer, Omron HJ-720 and Yamax Digiwalker SW-701 pedometer

For peer review only

step counts in lab-based and free-living settings. *J Sports Sci* 2015;**33**:991–1000. doi:10.1080/02640414.2014.981848

29 Trost SG, Mciver KL, Pate RR. Conducting Accelerometer-Based Activity Assessments in Field-Based Research: *Med Sci Sports Exerc* 2005;**37**:S531–43. doi:10.1249/01.mss.0000185657.86065.98

30 Jané-Llopis E, Matytsina I. Mental health and alcohol, drugs and tobacco: a review of the comorbidity between mental disorders and the use of alcohol, tobacco and illicit drugs. *Drug Alcohol Rev* 2006;**25**:515–36. doi:10.1080/09595230600944461

31 Babor TF, Higgins-Biddle JC, Saunders JB, *et al. AUDIT The Alcohol Use Disorders Identification Test. Guidelines for Use in Primary Care.* Second Edition. World Health Organization Department of Mental Health and Substance Dependence 2001.

32 Actigraph Software Department. *ActiLife 6 User's Manual*. Pensacola, FL: 2012.

33 Shek DTL, Ma CMS. Longitudinal Data Analyses Using Linear Mixed Models in SPSS: Concepts, Procedures and Illustrations. *Sci World J* 2011;**11**:42–76. doi:10.1100/tsw.2011.2

34 Brown H, Prescott R. *Applied mixed models in medicine*. Third edition. Chichester, West Sussex ; Hoboken: : John Wiley & Sons Inc 2015.

35 Hsueh Y. The Hawthorne experiments and the introduction of Jean Piaget in American industrial psychology, 1929-1932. *Hist Psychol* 2002;**5**:163–89. doi:10.1037//1093-4510.5.2.163

36 Paykel ES, Brugha T, Fryers T. Size and burden of depressive disorders in Europe. *Eur Neuropsychopharmacol* 2005;**15**:411–23. doi:10.1016/j.euroneuro.2005.04.008

37 Rubin DB. Inference and missing data. *Biometrika* 1976;**63**:581–92. doi:10.1093/biomet/63.3.581

38 Walters P, Barley EA, Mann A, *et al.* Depression in Primary Care Patients with Coronary Heart Disease: Baseline Findings from the UPBEAT UK Study. *PLoS ONE* 2014;**9**:e98342. doi:10.1371/journal.pone.0098342

39 Sundboll J, Schmidt M, Adelborg K, *et al.* Impact of pre-admission depression on mortality following myocardial infarction. *Br J Psychiatry* 2017;**210**:356–61. doi:10.1192/bjp.bp.116.194605

40 Bush DE, Ziegelstein RC, Tayback M, *et al.* Even minimal symptoms of depression increase mortality risk after acute myocardial infarction. *Am J Cardiol* 2001;**88**:337–41. doi:10.1016/S0002-9149(01)01675-7

41 Cooney GM, Dwan K, Greig CA, *et al.* Exercise for depression. 2013. doi:10.1002/14651858.cd004366.pub6

42 Chalder M, Wiles NJ, Campbell J, *et al.* Facilitated physical activity as a treatment for depressed adults: randomised controlled trial. *BMJ* 2012;**344**:e2758–e2758. doi:10.1136/bmj.e2758

For peer review only

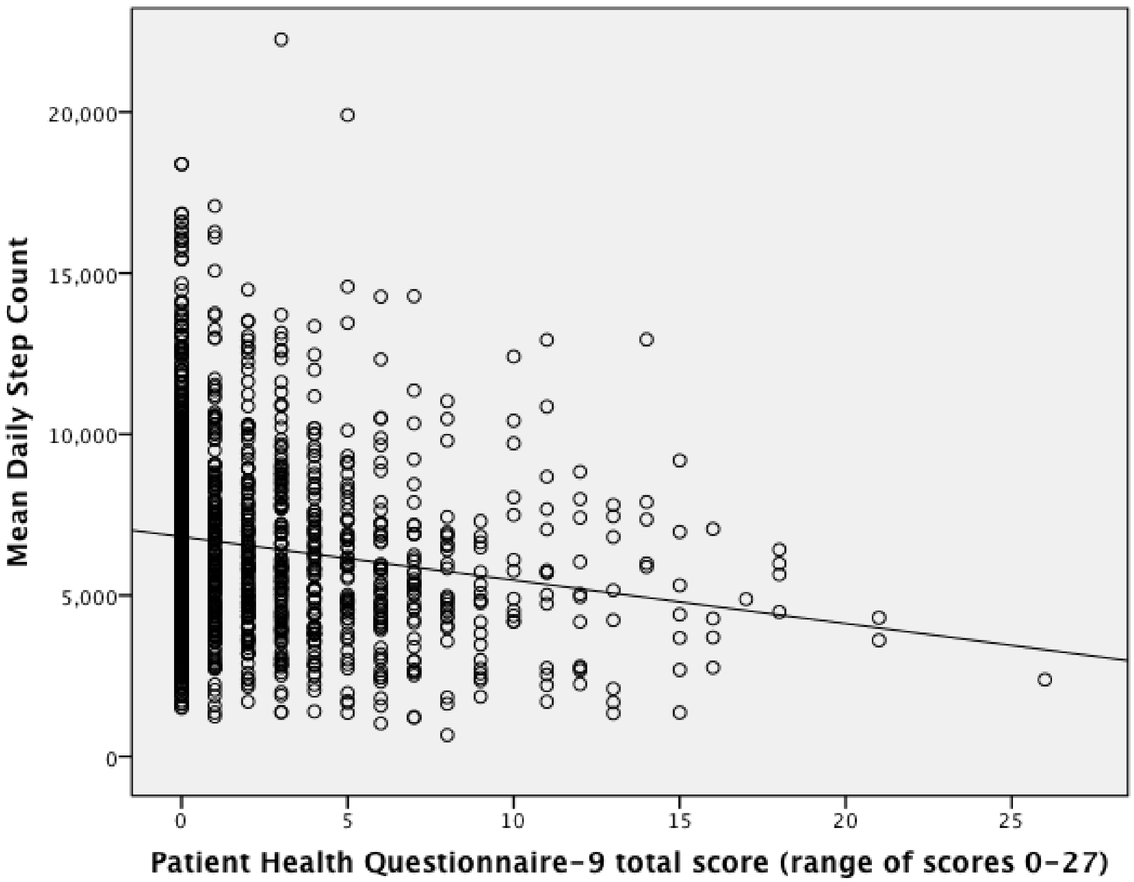

Figure 1: The association between depressive symptoms and daily step count at baseline in adults with high cardiovascular risk in a randomised controlled trial assessing the effectiveness of an intensive lifestyle intervention to improve physical activity and reduce weight.

Table 1: Estimated effects (coefficients and relative % change) of individual characteristics on average daily steps in subjects at high risk of CVD with ≥5 valid wear days

Variables	Model 1[a]		Model 2[b]	
	Coefficient	Relative % Change (95% CI)	Coefficient	Relative % Change (95% CI)
**Basic confounders constant**	8.834		8.942	
**Age** (impact of additional year)	0.002	0.2 (-0.3 to 0.7)	-0.013***	-1.3 (-1.8 to -0.7)
**Gender** Male	0[c]	0[c]	0[c]	0[c]
Female	-0.239***	-21.3 (-25.9 to -16.4)	-0.143***	-13.4 (-18.4 to -7.9)
**Day** Weekday	0[c]	0[c]	0[c]	0[c]
Weekend	-0.162***	-14.9 (-16.6 to -13.2)	-0.161***	-14.9 (-16.6 to -13.2)
**Season** Spring	0[c]	0[c]	0[c]	0[c]
Summer	-0.001	-0.1 (-6.1 to 6.3)	0.011	1.1 (-4.8 to 7.3)
Autumn	-0.005	-0.5 (-6.4 to 5.8)	-0.005	-0.5 (-6.2 to 5.6)
Winter	-0.095**	-9.0 (-14.4 to -3.3)	-0.102**	-9.7 (-14.9 to -4.2)
**a) Depressive Symptoms constant**	8.854		8.942	
None (PHQ-9: 0 - 4)	0[c]	0[c]	0[c]	0[c]
Mild (PHQ-9: 5 - 9)	-0.149***	-13.9 (-19.4 to -7.9)	-0.124***	-11.7 (-17.2 to -5.8)
Moderate/Severe (PHQ-9≥10)	-0.171**	-15.7 (-24.7 to -5.6)	-0.135**	-12.6 (-21.5 to -2.7)
**b) Ethnicity constant**	8.848		8.942	
White	0[c]	0[c]	0[c]	0[c]
Black/Asian/Other	-0.136***	-12.7 (-18.5 to -6.4)	-0.193***	-17.5 (-23.1 to -11.5)
**c) Education level constant**	8.872		8.942	
Level 3: A Level or higher	0[c]	0[c]	0[c]	0[c]
Level 2: O Level/GCSE/NVQ	-0.072**	-7.0 (-11.5 to -2.1)	-0.058*	-5.6 (-10.1 to -1.0)
Level 1: no formal qualification	-0.087**	-8.3 (-13.0 to -3.4)	-0.075**	-7.2 (-11.8 to -2.5)
**d) BMI constant**	8.830		8.942	
Impact of one unit BMI increase	-0.025***	-2.5 (-3.0 to -2.0)	-0.028***	-2.8 (-3.3 to -2.3)
**e) Smoking status constant**	8.863		8.942	
Never smoked	0[c]	0[c]	0[c]	0[c]
Ex-smoker	-0.016	-1.5 (-6.1 to 3.3)	-0.027	-2.7 (-7.1 to 2.0)
Current smoker	-0.120**	-11.3 (-17.2 to -5.0)	-0.189***	-17.2 (-22.6 to -11.4)
**f) Drinking of Alcohol constant**	8.844		8.942	
Low risk (AUDIT: 1-7)	0[c]	0[c]	0[c]	0[c]
Possibly harmful (AUDIT≥8)	0.004	0.4 (-5.3 to - 6.5)	0.035	3.6 (-2.1 to 9.7)
Abstainer (AUDIT: 0	-0.119**	-11.3 (-17.3 to -4.8)	-0.089*	-8.6 (-14.7 to -1.9)

*[a]Model 1: all variables adjusted for basic confounders (age, gender, day, season). [b]Model 2: all variables mutually adjusted. [c]Reference group is set to 0. Significance levels: *<.05, **<.01, ***<.001.*

For peer review only

Table 2: Estimated effects (coefficients and relative % change) of individual characteristics on average daily steps in subjects at high risk of CVD with a QRisk2 ≥ 20%

Variables		Model 1[a]		Model 2[b]	
		Coefficient	Relative % Change (95% CI)	Coefficient	Relative % Change (95% CI)
**Basic confounders constant**		8.803		8.918	
**Age** (impact additional year)		0.004***	0.4 (-0.1 to 1.0)	-0.011***	-1.1 (-1.7 to -0.5)
**Gender**	Male	0[c]	0[c]	0[c]	0[c]
	Female	-0.225***	-20.1 (-25.2 to -14.7)	-0.133***	-12.4 (-18.0 to -6.5)
**Day**	Weekday	0[c]	0[c]	0[c]	0[c]
	Weekend	-0.162***	-15.0 (-16.7 to -13.2)	-0.162***	-15.0 (-16.7 to -13.1)
**Season**	Spring	0[c]	0[c]	0[c]	0[c]
	Summer	-0.002	-0.3 (-6.4 to 6.4)	0.0128	1.3 (-4.8 to 7.8)
	Autumn	-0.015	-1.5 (-7.6 to 5.0)	-0.018	-1.8 (-7.6 to 4.5)
	Winter	-0.094**	-9.0 (-14.6 to -3.0)	-0.103**	-9.8 (-15.2 to -4.0)
**a) Depressive Symptoms constant**		8.830		8.918	
None (PHQ-9: 0 - 4)		0[c]	0[c]	0[c]	0[c]
Mild (PHQ-9: 5 - 9)		-0.177***	-16.2 (-21.7 to -10.4)	-0.148***	-13.8 (-19.3 to -7.9)
Moderate/Severe (PHQ-9≥10)		-0.234***	-20.9 (-29.3 to -11.5)	-0.206***	-18.6 (-26.9 to -9.3)
**b) Ethnicity constant**		8.817		8.918	
White		0[c]	0[c]	0[c]	0[c]
Black/Asian/Other		-0.133***	-12.4 (-18.6 to -5.7)	-0.181***	-16.6 (-22.6 to -10.1)
**c) Education level constant**		8.835		8.918	
A Level or higher		0[c]	0[c]	0[c]	0[c]
O Level/GCSE/NVQ		-0.059*	-5.7 (-10.6 to -0.6)	-0.047	-4.6 (-9.4 to -0.4)
No formal qualification		-0.073**	-7.0 (-11.9 to -1.9)	-0.056*	-5.5 (-10.3 to -0.4)
**d) BMI constant**		8.804		8.918	
BMI per one unit increase		-0.023***	-2.3 (-2.8 to -1.8)	-0.026***	-2.6 (-3.1 to -2.1)
**e) Smoking status constant**		8.835		8.918	
Never smoked		0[c]	0[c]	0[c]	0[c]
Ex-smoker		-0.015	-1.5 (-6.4 to 3.7)	-0.028	-2.8 (-7.5 to 2.2)
Current smoker		-0.139***	-12.9 (-19.0 to -6.5)	-0.203***	-18.4 (-24.0 to -12.3)
**f) Drinking of Alcohol constant**		8.810		8.918	
Low risk (AUDIT: 1-7)		0[c]	0[c]	0[c]	0[c]
Possibly harmful (AUDIT≥8)		0.011	1.1 (-4.9 to 7.5)	0.042	4.25 (-1.7 to 10.7)
Abstainer (AUDIT: 0)		-0.095*	-9.1 (-15.6 to -2.1)	-0.072	-7.0 (-13.6 to 0.2)

*[a]Model 1: all variables adjusted for basic confounders (age, gender, day, season). [b]Model 2: all variables mutually adjusted. [c]Reference group is set to 0. Significance levels: *<.05, **<.01, ***<.001.*

For peer review only

STROBE 2007 (v4) Statement—Checklist of items that should be included in reports of *cross-sectional studies*

Section/Topic	Item #	Recommendation	Reported on page #
**Title and abstract**	1	(*a*) Indicate the study's design with a commonly used term in the title or the abstract	1
		(*b*) Provide in the abstract an informative and balanced summary of what was done and what was found	2
**Introduction**			
Background/rationale	2	Explain the scientific background and rationale for the investigation being reported	3-5
Objectives	3	State specific objectives, including any prespecified hypotheses	4-5
**Methods**			
Study design	4	Present key elements of study design early in the paper	5
Setting	5	Describe the setting, locations, and relevant dates, including periods of recruitment, exposure, follow-up, and data collection	5-7
Participants	6	(*a*) Give the eligibility criteria, and the sources and methods of selection of participants	5
Variables	7	Clearly define all outcomes, exposures, predictors, potential confounders, and effect modifiers. Give diagnostic criteria, if applicable	5-7
Data sources/ measurement	8*	For each variable of interest, give sources of data and details of methods of assessment (measurement). Describe comparability of assessment methods if there is more than one group	5-7
Bias	9	Describe any efforts to address potential sources of bias	8-9
Study size	10	Explain how the study size was arrived at	9
Quantitative variables	11	Explain how quantitative variables were handled in the analyses. If applicable, describe which groupings were chosen and why	5-7
Statistical methods	12	(*a*) Describe all statistical methods, including those used to control for confounding	7-9
		(*b*) Describe any methods used to examine subgroups and interactions	-
		(*c*) Explain how missing data were addressed	7
		(*d*) If applicable, describe analytical methods taking account of sampling strategy	-
		(*e*) Describe any sensitivity analyses	8-9

For peer review only

**Results**			
Participants	13*	(a) Report numbers of individuals at each stage of study—eg numbers potentially eligible, examined for eligibility, confirmed eligible, included in the study, completing follow-up, and analysed	9
		(b) Give reasons for non-participation at each stage	9
		(c) Consider use of a flow diagram	-
Descriptive data	14*	(a) Give characteristics of study participants (eg demographic, clinical, social) and information on exposures and potential confounders	9-11
		(b) Indicate number of participants with missing data for each variable of interest	9-11
Outcome data	15*	Report numbers of outcome events or summary measures	9-10
Main results	16	(*a*) Give unadjusted estimates and, if applicable, confounder-adjusted estimates and their precision (eg, 95% confidence interval). Make clear which confounders were adjusted for and why they were included	12-13
		(*b*) Report category boundaries when continuous variables were categorized	6-9
		(*c*) If relevant, consider translating estimates of relative risk into absolute risk for a meaningful time period	-
Other analyses	17	Report other analyses done—eg analyses of subgroups and interactions, and sensitivity analyses	8-9
**Discussion**			
Key results	18	Summarise key results with reference to study objectives	14
Limitations	19	Discuss limitations of the study, taking into account sources of potential bias or imprecision. Discuss both direction and magnitude of any potential bias	14-15
Interpretation	20	Give a cautious overall interpretation of results considering objectives, limitations, multiplicity of analyses, results from similar studies, and other relevant evidence	15-17
Generalisability	21	Discuss the generalisability (external validity) of the study results	16-17
**Other information**			
Funding	22	Give the source of funding and the role of the funders for the present study and, if applicable, for the original study on which the present article is based	18

*Give information separately for cases and controls in case-control studies and, if applicable, for exposed and unexposed groups in cohort and cross-sectional studies.

Note: An Explanation and Elaboration article discusses each checklist item and gives methodological background and published examples of transparent reporting. The STROBE checklist is best used in conjunction with this article (freely available on the Web sites of PLoS Medicine at http://www.plosmedicine.org/, Annals of Internal Medicine at http://www.annals.org/, and Epidemiology at http://www.epidem.com/). Information on the STROBE Initiative is available at www.strobe-statement.org.

For peer review only

BMJ Open

The association between depressive symptoms and objectively measured daily step count in individuals at high risk of cardiovascular disease in south London, United Kingdom: a cross-sectional study

Journal:	*BMJ Open*
Manuscript ID	bmjopen-2017-020942.R1
Article Type:	Research
Date Submitted by the Author:	30-Jan-2018
Complete List of Authors:	Ludwig, Vera; Technische Universitat Dresden Medizinische Fakultat Carl Gustav Carus, Department of Medicine III<br>Bayley, Adam; Institute of Psychiatry, Psychology and Neuroscience, King's College London, Department of Psychological Medicine<br>Cook, Derek; St George's University, Population Health Research Institute<br>Stahl, Daniel; Institute of Psychiatry, Psychology and Neuroscience, King's College London, Department of Biostatistics and Health Informatics<br>Treasure, Janet; Institute of Psychiatry, Psychology and Neuroscience, King's College London, Section of Eating Disorders<br>Asthworth, Mark; King's College London, UK, Primary Care and Public Health Sciences<br>Greenough, Anne; Kings College London, Department of Women and Children's Health, School of Life Sciences, Faculty of Life Science and Medicine; King's College London, 7MRC & Asthma UK Centre in Allergic Mechanisms of Asthma<br>Winkley, Kirsty; Institute of Psychiatry, Psychology and Neuroscience, King's College London, Department of Psychological Medicine<br>Bornstein, Stefan ; University Hospital Carl Gustav Carus, Technical University Dresden, Department of Medicine III<br>Ismail, Khalida; Institute of Psychiatry, Psychology and Neuroscience, King's College London, Department of Psychological Medicine
**Primary Subject Heading**:	Sports and exercise medicine
Secondary Subject Heading:	Mental health, Public health, Cardiovascular medicine
Keywords:	Adult psychiatry < PSYCHIATRY, PUBLIC HEALTH, SPORTS MEDICINE

SCHOLARONE™
Manuscripts

The association between depressive symptoms and objectively measured daily step count in individuals at high risk of cardiovascular disease in south London, United Kingdom: a cross-sectional study

Vera M. Ludwig[1], Adam Bayley[2], Derek G. Cook[3], Daniel Stahl[4], Janet L. Treasure[5], Mark Ashworth[6], Anne Greenough[7-9], Kirsty Winkley[2], Stefan R. Bornstein[1], Khalida Ismail[2]

[1]Department of Medicine III, University Hospital Carl Gustav Carus, Technical University Dresden, Germany.

[2]Department of Psychological Medicine, Institute of Psychiatry, Psychology and Neuroscience, King's College London, UK.

[3]Population Health Research Institute, St George's, University of London, UK.

[4]Department of Biostatistics and Health Informatics, Institute of Psychiatry, Psychology and Neuroscience, King's College London, UK.

[5]Section of Eating Disorders, Institute of Psychiatry, Psychology and Neuroscience, King's College London, UK.

[6]Department of Primary Care and Public Health Sciences, King's College London, UK.

[7]MRC & Asthma UK Centre in Allergic Mechanisms of Asthma, King's College London.

[8]Department of Women and Children's Health, School of Life Course Sciences, Faculty of Life Sciences and Medicine, Kings College London.

[9]NIHR Biomedical Research Centre at Guy's and St Thomas' NHS Foundation Trust and King's College London.

Corresponding author: Khalida Ismail, Department of Psychological Medicine, King's College London, 10 Cutcombe Road, London SE5 9RJ, UK. Tel: +44 (0)20 7848 5131. E-mail: khalida.2.ismail@kcl.ac.uk

Word count: 4,336 words

Key words: physical activity, accelerometer, PHQ-9, QRisk2, CVD

For peer review only

ABSTRACT

Objectives: Depressive symptoms are common, but rarely considered a risk factor for unhealthy lifestyles associated with cardiovascular disease (CVD). This study investigates whether depressive symptoms are associated with reduced physical activity (PA) in individuals at high risk of developing CVD.

Design: Secondary analysis of the cross-sectional baseline data from a randomised controlled trial of an intensive lifestyle intervention.

Setting: 135 primary care practices in south London, UK.

Participants: 1,742 adults, 49-74 years, 86% male at high (≥20%) risk of developing CVD in the next 10 years as defined via QRisk2-Score.

Outcome measures: The main explanatory variable was depressive symptoms measured via the Patient Health Questionnaire-9 (PHQ-9). The main outcome was daily step count measured with an accelerometer (ActiGraph GT3X) stratified by weekdays and weekend days.

Results: The median daily step count of the total sample was 6,151 (IQR, 3,510) with significant differences ($p<.001$) in mean daily step count between participants with low (PHQ-9 score 0 to 4), mild (PHQ-9 score 5 to 9), and moderate to severe depressive symptoms (PHQ-9 score ≥10). Controlling for age, gender, ethnicity, education level, body mass index (BMI), smoking, consumption of alcohol, day of the week and season, individuals with mild depressive symptoms and those with moderate to severe depressive symptoms walked 13.3% (CI 18.8 to 7.9) and 15.6% (CI 23.7 to 6.5) less than non-depressed individuals respectively. Furthermore, male gender, white ethnicity, higher education level, lower BMI, non-smokers, moderate alcohol intake, weekdays, and summer season were independently associated with higher step count.

Conclusions: People at high risk of CVD with depressive symptoms have lower levels of physical activity.

Trial registration: ISRCTN84864870

ARTICLE SUMMARY

Strengths and limitations of this study

- This study is the first to look at the relationship between depressive symptoms and objectively measured physical activity in a large sample (n=1,720) of individuals at high risk of developing CVD.
- The analysis controls for potential confounding of: age, gender, ethnicity, education level, BMI, smoking, consumption of alcohol, day of the week, and season.
- The inclusion of participants with as little as one valid wear day tries to minimise bias by not excluding more depressed participants that did not wear the monitor the requested five days.
- No causalities can be inferred from the cross-sectional data.
- As it is a sample from the general population, the proportion of severely depressed subjects is small, and the power to compare the severity categories of depressive symptoms on step count is limited.

INTRODUCTION

Cardiovascular disease (CVD) remains the most common cause of death worldwide: it accounts for a third of global mortality[1] and contributes considerably to years lost to disability.[2] The main lifestyle risk factors for CVD are tobacco smoking, physical inactivity, unhealthy diets and the harmful use of alcohol.[1] Estimations suggest that increasing physical activity (PA) to the World Health Organisation recommended amount would reduce the global CVD related burden by 6%.[3] It is increasingly recognised that depression (defined here as an umbrella term for depressive disorder and depressive symptoms) is an independent risk factor for CVD[4] and is associated with increased risk of mortality after a CVD event,[5] but mental health is not routinely considered as a factor that may affect uptake of PA to improve CVD outcomes in clinical practice or intervention studies.

For peer review only - http://bmjopen.bmj.com/site/about/guidelines.xhtml

For most individuals and especially the elderly, the most accessible and safest form of PA is walking. Increased number of daily steps, independent of walking speed or intensity, is associated with improved biomedical outcomes such as lower body mass index (BMI),[6] lower cholesterol ratio and triglyceride levels,[7] and a lower rate of CVD incidents overall.[8] Self-report questionnaires to assess walking are prone to over and under reporting biases.[9] Using objective tools to assess walking, such as accelerometers or pedometers, can overcome this limitation.[10] Potential confounders of any association between depressive symptoms and walking include younger age, male gender, lower BMI and white ethnicity, which are associated with higher step count.[11–14] Education is also a potential confounder although there have been mixed findings.[11,12,15] There are also well known time effects namely that people walk more on weekdays than on weekends and during summer months than in winter.[14]

The relationship between depressive symptoms and step count have only been assessed in specific populations with small sample sizes, such as low-socioeconomic status Latino immigrants,[16] elderly Japanese people[17] or patients with chronic conditions such as heart failure,[18,19] or chronic obstructive pulmonary disease.[20,21] Studies yield contradictory results, with some observing no association between depressive symptoms and daily step count,[19,21] while others report a negative correlation.[16–18,20] In one cross sectional sample of healthy older adults, an inverse association between depressive symptoms (using the Goldberg Depression Scale-15) and accelerometer measured daily step count disappeared after controlling for general health and disability.[22] While a systematic review suggests reduced levels of objectively measured PA in depressed patients,[23] it is not known whether this association is present in those at high risk of CVD and taken into account important confounding such as gender and age.

For peer review only

We aim to describe the distribution of objectively measured average daily step count in a sample at high risk of CVD and to test the hypothesis that individuals with greater depressive symptoms are likely to have reduced daily step counts, controlling for potential confounding.

METHODS AND MATERIAL

Design

This is a cross-sectional analysis of the baseline data of individuals at high risk of CVD who were participating in a randomised controlled trial (RCT) testing the effectiveness of an intensive lifestyle intervention 'MOtiVational intErviewing InTervention' (MOVE IT) to increase PA and reduce weight. The data were collected before participants were randomised.

Setting and sampling frame

The MOVE IT study was conducted in the 12 south London Clinical Commissioning Groups (CCGs), which are the local administrative authorities for commissioning health care for its residents, representing a diverse socioeconomic and ethnic population of approximately three million UK residents. All primary care practices with a list size of >5,000 registered patients, resident within these CCGs were invited to participate. Within these practices, patients with a ≥20% risk of having a fatal or non-fatal CVD event in the next 10 years as defined by the QRisk2 score (2013 version),[24] were identified from the practice register. The QRisk2 score is a validated predictive tool for identifying individuals at risk of developing a fatal or non-fatal cardiovascular event in the next 10 years based on age, smoking status, ethnicity, systolic blood pressure, ratio of total serum cholesterol to high density lipoprotein (HDL) cholesterol, BMI, family history of coronary heart disease in first degree relative, Townsend deprivation score, treated hypertension and diagnosis of rheumatoid arthritis.[24] Further patient inclusion criteria were: age ≥40 and ≤74 years; permanent residency and fluent in English. The exclusion criteria were: known CVD; on the general practice electronic register for diabetes, chronic kidney disease, atrial fibrillation or stroke; chronic obstructive pulmonary disease or morbid obesity (BMI>50kg/m^2). Participants were recruited from June

For peer review only

7th 2013 to February 19th 2015. Ethical approval was granted by the Dulwich Ethics Committee (reference: 12/LO/0917) and all participants gave written informed consent. Further details of the trial protocol are available.[25]

Measures

The main explanatory variable was self-report depressive symptoms using the Patient Health Questionnaire-9 (PHQ-9).[26] The PHQ-9 scores the occurrence of each of the nine Diagnostic and Statistical Manual of Mental Disorders 4th Edition (DSM-IV) criteria for major depressive disorder over the past two weeks on a scale from 0 (not at all) to 3 (nearly every day). The total sum score, ranging from 0 to 27, can be categorised as not depressed (0–4), mild (5–9), moderate (10–14), moderately severe (15–19), and severe depression (20–27).[26] A score of ten or above defined as clinically relevant depression and a recent meta-analysis reported that a cut-off score ≥10 generated a sensitivity of 77% and specificity of 85% in diagnosis for major depression.[27] In our study, the number of individuals with scores in the three top categories (10–14, 15–19 and 20–27) was low (2.9, 0.9 and 0.2% respectively), therefore we merged them to create one category of moderate to severe 'clinical' depression (score ≥10).

The main outcome was the average daily number of steps measured with the ActiGraph GT3X accelerometer (ActiGraph, Florida, United States), a tri-axial movement sensor that has been validated on number of steps taken and is reported to record 98.5% of steps.[28] Number of steps correlated (r=.70) with average moderate-to-vigorous physical activity (MVPA). We chose steps per day as the main outcome measure in this analysis as it was specified as the main outcome measure in the MOVE IT trial and walking is the main form of physical activity in this elderly population. We also note that steps per day was highly correlated with minutes of MVPA (r=.75). Steps also has the merit that is an easily understood measure which facilitates clinical application of the results. The accelerometers were given to the participants by a researcher who advised them to attach the monitor to the

For peer review only - http://bmjopen.bmj.com/site/about/guidelines.xhtml

waist and wear it for seven days from waking in the morning until going to bed at night, only removing it for bathing and swimming. If monitors were worn for less than nine hours (540 minutes) per day, these wear days were considered missing as it suggests the participant forgot to wear the monitor for a part of the day. The aim was to generate a minimum of five valid wear days per participant to ensure the collected data represents habitual activity levels.[29] A preliminary analysis revealed that people who wore the monitor for less than five days exhibited significantly more depressive symptoms that those who wore it for at least five days so to reduce bias we included individuals who had less than five days of wear time. We therefore included all participants with at least one valid wear day in the analysis and performed a sensitivity analysis to determine whether including individuals with one wear day only altered the results.

To account for potential confounding, reported covariates of depressive symptoms and objectively measured PA (age, gender, BMI, ethnicity, day of the week, level of education,[11] season,[14] smoking and alcohol intake[30]) were controlled for in the analysis. During the one-on-one interview with the researcher, participants reported age, gender and ethnicity and were weighed and measured with standardized scales.[25] Self-reported levels of educational attainment were categorised into: level 1: no formal qualifications; level 2: qualifications at the end of compulsory school education in UK typically at age 16 years; or level 3: qualifications awarded at the end of further education typically at age 18 years. Reported smoking status was categorised as: never smoked, ex-smoker and current smoker. Alcohol Use Disorders Identification Test (AUDIT), a 10-item structured questionnaire that categorises alcohol consumption, grouped participants into abstainers (AUDIT score= 0), low risk (score 1-7) and possibly hazardous or harmful drinkers (score ≥8).[31]

Statistical Analysis

For peer review only - http://bmjopen.bmj.com/site/about/guidelines.xhtml

Data on daily step count were downloaded from the activity monitors using Actilife Version 6.11.7.[32] The number of valid wear days (≥540 minutes) per participant was counted. Extreme outliers in daily step count were individually investigated to ensure plausibility and rule out device failure. This was not the case and no cases were removed from the analysis. Measurements of step count were divided into weekdays (Monday to Friday) and weekend days (Saturday, Sunday). Within these categories, the arithmetic mean of the step count for each participant was calculated, thereby creating at least one and at most two measurements per participant. Baseline characteristics were summarised as means (± standard deviation) or percentages. As the step count data were not normally distributed, median and its interquartile range (IQR) are presented and non-parametric Spearman rank correlations were applied. To identify relevant confounders of step count, Mann-Whitney U and Kruskall Wallis tests were conducted to compare differences between categories of potential confounders. No adjustments for multiple testing were made in order avoid missing potentially important confounders due to an inflation of the type II error for our final model.[33]

For the main analysis, a linear mixed model (LMM) was used. LMMs can account for the dependency of repeated observations of the same subject (weekdays and weekends) and allow including all individuals with at least one outcome observation.[34] To improve the fit of the model, the step count data were log-transformed before they were entered into the model. In order to investigate the sizes of the adjusted and unadjusted effects of each predictor on step count, we introduced two models. The first model aims to describe the individual associations between each predictor and mean daily step count. We controlled for age, gender, season and day of accelerometer wear (basic confounders) and then individually entered each additional covariate, a) depressive symptoms, b) ethnicity, c) education level, d) BMI, e) smoking status and f) drinking of alcohol, into the LMM as independent predictor for log step count, obtaining the estimated change caused by each of the predictors. In the second model, all variables were entered simultaneously to determine

For peer review only - http://bmjopen.bmj.com/site/about/guidelines.xhtml

the mutually adjusted effects of all predictors. Age and BMI were entered as continuous fixed effects variables (centred around the mean) and the others were entered as categorical fixed effects variables. We used an unstructured covariance structure for the LMM. Assumptions of the LMM were assessed by visual inspection of the residual plots.[35] Controlling for nesting at practice level showed that the estimated variance was virtually 0, thus practice level was excluded from the final LMM. The constant for each LMM is reported alongside the coefficients of relative change for each variable. These relative differences between the log transformed step counts were back transformed to the percentage difference in step count by applying the formula $100(e^{\text{Coefficient}}-1)$. To account for multiple testing in the final LMM, the alpha-level is lowered to α=.01 and predictors with p-values <.05, but >.01 are treated as trend and interpreted with caution.[36]

A sensitivity analysis was conducted to determine if wearing the accelerometer for less than five days alters the results by rerunning the main analyses on participants with at least five wear days (Supplementary Table 1). Re-analysis of QRisk2 scores using cleaned data and the QRisk2 2013 batch calculator revealed that 155 (9%) of the included participants had a QRisk2 score <20%. We conducted a second sensitivity analysis by rerunning the main analyses including only patients with a QRisk2 score of ≥20 (Supplementary Table 2) to check for potential bias. Independent sample t-tests were used to compare depression scores between subsamples (≥5 vs. <5 wear days and ≥20% and <20% QRisk2). Data were analysed using IBM SPSS Statistics for Macintosh, Version 24.0 (SPSS Inc., Chicago, IL) was used for all analyses.

RESULTS

Population Characteristics

The MOVE IT sample consisted of 135 primary care practices in the 12 south London CCGs, from which 1,742 participants were eligible on screening and consented to participate. At baseline, only n=22 (1.3%) provided less than one valid wear day with ≥540 minutes and had

to be excluded right away. Additionally, 117 participants (6.7%) provided at least one, but less than the requested five full wear days. When comparing these 117 participants with those who wore the monitor for five days or more, we found that the former were significantly more depressed than the latter (p<.001). If we excluded this group, this may lead to an underestimation of the effect of depression on step count therefore we decided to include this group, giving a final sample of 1,720. The average QRisk2 score of the sample was 25% (SD 4.9). On re-calculation of QRisk2 scores, 155 patients with a score <20% had been recruited into the trial. As there was no difference in depressive symptoms compared to those with QRisk2 score ≥20% (p=.394), we retained this subgroup in the analysis. The mean age was 69 (SD 4.1) years, the vast majority were male, and of white ethnicity. Demographic and behavioural characteristics of the final sample stratified by accelerometer wear day are reported in Table 1.

Step count data

A total of 11,140 wear days from n=1,720 participants were included in the analysis. These participants provided a mean of 6.5 (SD 1.2) wear days. Daily step count ranged from 105 to 21,313 steps with a median of 6,151 (IQR 3,510). Figure 1 shows an inverse relationship (Spearman's rho r=-.149, p=.01) between depressive symptom score and mean individual daily step count. Step count also differs between categories of gender, ethnicity, education level, BMI, smoking, drinking of alcohol, and with weekdays, but not age (Table 1).

For peer review only

Table 1 Demographic characteristics and distribution of median daily step count in a randomised controlled trial assessing the effectiveness of an intensive lifestyle intervention

Variables		Weekdays			Weekends		
		N (%)	Median Steps (IQR)[a]	p-value[b]	N (%)	Median Steps (IQR)[a]	p-value[b]
**Total study population**		1,719 (100)	6,447 (3,875)		1,645 (100)	5,676 (3,737)	
**Age**	>70	58 (3.4)	6,434 (4,175)	p=.859	52 (3.2)	5,639 (4,209)	p=.454
	50-59[c]	701 (40.8)	6,319 (4,017)		661 (40.2)	5,784 (3,985)	
	60-69	960 (55.8)	6,485 (3,738)		932 (56.7)	5,640 (3,659)	
**Gender**	Male	1,470 (85.5)	6,665 (3,910)	p<.001	1,402 (85.2)	5,903 (3,870)	p<.001
	Female	249 (14.5)	5,441 (3,343)		243 (14.8)	4,590 (3,143)	
**Ethnicity**	White	1,538 (89.5)	6,536 (3,895)	p<.001	1,472 (89.5)	5,800 (3,757)	p<.001
	Black/Asian/Other	181 (10.5)	5,619 (3,493)		173 (10.5)	4,676 (3,321)	
**Education level[d]**	Level1	427 (25.3)	6,022 (3,742)	p=.001	399 (24.7)	5,287 (3,713)	p=.018
	Level 2	467 (27.6)	6,391 (4,070)		444 (27.5)	5,517 (3,907)	
	Level 3	796 (47.1)	6,734 (3,871)		774 (47.9)	5,928 (3,690)	
**Depressive symptoms (PHQ-9)**	None (0-4)	1,449 (84.3)	6,656 (3,855)	p<.001	1,394 (84.7)	5,804 (3,831)	p<.001
	Mild (5-9)	201 (11.7)	5,563 (3,330)		189 (11.5)	5,045 (3,473)	
	Mod./Sev. (≥10)	69 (4.0)	5,428 (3,905)		62 (3.8)	5,258 (3,939)	
**BMI (kg/m^2)**	<20	24 (1.4)	6,899 (4,774)	p<.001	22 (1.3)	6,178 (3,899)	p<.001
	20-24.9	371 (21.6)	7,255 (3,983)		365 (22.2)	6,307 (4,329)	
	25-29.9	800 (46.5)	6,550 (3,736)		773 (47.0)	5,796 (3,615)	
	≥30	524 (30.5)	5,850 (3,594)		485 (29.5)	5,108 (3,559)	
**Smoking status**	Never smoked	482 (28.0)	6,485 (3,694)	p<.001	468 (28.4)	5,795 (3,760)	p=.047
	Ex-Smoker	975 (56.7)	6,605 (3,850)		929 (56.5)	5,784 (3,670)	
	Current Smoker	262 (15.2)	5,795 (4,285)		248 (15.1)	5,268 (4,115)	
**Alcohol intake (AUDIT)**	Abstainer (0)	176 (10.2)	5,533 (4,208)	p<.001	171 (10.4)	4,984 (3,598)	p=.001
	Low risk (1-7)	1,274 (74.2)	6,544 (3,628)		1,216 (73.9)	5,767 (3,629)	
	Harmful (≥8)	269 (15.6)	6,490 (4,370)		258 (15.7)	5,787 (4,027)	

[a]Median step count and IQR are rounded to the closest full number. [b]p-values are calculated with Mann-Whitney U tests and Kruskall Wallis tests. [c]One person was younger than 50 years (49), this person was included in the ≥50 category. [d]N=29 missing for weekdays, n=28 missing for weekends. PHQ-9: Patient Health Questionnaire-9. AUDIT*:* Alcohol Use Disorders Identification.

Linear mixed model

The LMMs (Table 2) observed significant associations between depressive symptoms and step count. Controlling for age, gender day and season (Model 1), individuals with moderate to severe depressive symptoms walked 19.0% less (p<.001) than non-depressed individuals, and mildly depressed individuals walked 16.2% less (p<.001). Mutually controlling for all predictors (Model 2) decreased the effect of depressive symptoms to 15.6% and 13.5% respectively (p=.001, p<.001). All other covariates influenced PA as well. In Model 2, each additional year in age and one point increase in BMI was associated with a decrease in step count by 1.2% and 2.7% respectively (p<.001, p<.001). Female gender reduced step count by 13.8% (p<.001), individuals from ethnic minorities walked 17.8% less steps (p<.001). Level 3 qualifications tended to be associated with higher step count of 5.3 (Level 2, p=.028) and 5.7% (no formal qualifications p=.022). Current smokers' step count was 19.1% lower compared to people who had never smoked (p<.001) and participants abstaining from alcohol consumption had an 8.8% lower step count than people who are low risk drinkers (p=.01). On weekends 14.8% fewer steps were recorded than on weekdays (p<.001) and during the winter months there was a decrease of 9.3% compared to spring (p=.001). Sensitivity analyses revealed that rerunning the models with participants with at least 5 days of complete wear time (Supplementary Table 1) and participants with QRisk2 score >20 only (Supplementary Table 2), provided similar parameter estimates and did not alter the conclusions of this study.

For peer review only

Table 2 Estimated effects (coefficients and relative % change) of individual characteristics on average daily steps in subjects at high risk of CVD

Variables		Model 1[a] Coefficient	Model 1[a] Relative % Change (95% CI)	Model 2[b] Coefficient	Model 2[b] Relative % Change (95% CI)
**Constant**		8.813		8.937	
**Basic confounders**					
**Age** (impact additional year)		0.004	0.4 (-0.1 to 1.0)	-0.012***	-1.2 (-1.7 to -0.6)
**Gender**	Male	0	0	0	0
	Female	-0.249***	-22.1 (-26.7 to -17.2)	-0.149***	-13.8 (-18.9 to -8.5)
**Day**	Weekday	0	0	0	0
	Weekend	-0.161***	-14.9 (-16.6 to -13.2)	-0.161***	-14.9 (-16.6 to -13.1)
**Season**	Spring	0	0	0	0
	Summer	-0.007	-0.7 (-6.7 to 5.7)	0.004	0.4 (-5.4 to 6.7)
	Autumn	-0.013	-1.3 (-7.2 to 5.0)	-0.015	-1.5 (-7.2 to 4.5)
	Winter	-0.086**	-8.3 (-13.8 to -2.4)	-0.098**	-9.3 (-14.5 to -3.8)
**a) Depressive Symptoms constant**		8.840			
None (PHQ-9: 0 - 4)		0	0	0	0
Mild (PHQ-9: 5 - 9)		-0.177***	-16.2 (-21.5 to -10.6)	-0.142***	-13.3 (-18.6 to -7.6)
Moderate/Severe (PHQ-9≥10)		-0.211***	-19.0 (-27.2 to -10.0)	-0.170**	-15.6 (-23.8 to -6.6)
**b) Ethnicity constant**		8.845			
White		0	0	0	0
Black/Asian/Other		-0.145***	-13.5 (-19.2 to -7.4)	-0.196***	-17.8 (-23.3 to -12.0)
**c) Education level constant**		8.845			
Level 3: A Level or higher		0	0	0	0
Level 2: O Level/GCSE/NVQ		-0.063*	-6.1 (-10.7 to -1.2)	-0.054*	-5.3 (-9.7 to -0.6)
Level 1: no formal qualification		-0.072**	-7.0 (-11.7 to -2.0)	-0.196*	-5.7 (-10.4 to -0.9)
**d) BMI constant**		8.811			
Impact of one unit BMI increase		-0.025***	-2.4 (-2.9 to -1.9)	-0.027***	-2.7 (-3.2 to -2.2)
**e) Smoking status constant**		8.849			
Never smoked		0	0	0	0
Ex-smoker		-0.023	-2.3 (-6.9 to -2.5)	-0.038	-3.7 (-8.2 to 0.9)
Current smoker		-0.141***	-13.2 (-18.9 to -7.0)	-0.212***	-19.1 (-24.4 to -13.4)
**f) AUDIT score constant**		8.824			
Low risk (score 1-7)		0	0	0	0
Possibly harmful (score ≥8)		-0.001	-0.1 (-5.8 to 6.0)	0.032	3.3 (-2.4 to 9.3)
Abstainer (score 0)		-0.124**	-11.7 (-17.6 to -5.2)	-0.092*	-8.8 (-14.9 to -2.2)

[a]*Model 1: all variables adjusted for basic confounders (age, gender, day, season). Note: There is a constant for the basic confounders only model and separate constants for this model adjusted for each additional confounder (a-f).* [b]*Model 2: all variables mutually adjusted, thus there is only one constant for the whole model. Reference group within each category is set to 0. AUDIT:* Alcohol Use Disorders Identification Test. *Sign. levels: *<.05, **<.01, ***<.001.*

DISCUSSION

Main study findings

This study assessed patterns of PA and depressive symptoms in people at high risk of CVD. We found an inverse relationship where increased depressive symptoms were associated with significantly decreased step counts. Female gender, ethnic minorities, lower education level, higher BMI, smoking, alcohol abstainers, weekends and colder season were independently associated with lower daily step count.

Study strengths and limitations

The strengths of our study are, firstly, the large sample size. Furthermore, PA was objectively assessed based on accelerometer data. Socio-demographic, biomedical and depression data were collected in face-to-face interviews with trained researchers using standardised schedules, resulting in very few missing data. Recruitment was conducted year-round so seasonal variations were accounted for.

The limitations were that the study design was cross-sectional, so causal relationships cannot be inferred and it remains unclear whether people with depressive symptoms are at increased risk of walking less or less active people are at increased risk for depressive symptoms. Although PA was measured objectively using accelerometers, we cannot rule out that the recorded levels of physical activity are subject to bias, as participants might have changed their performance because they knew they were being observed.[37] This would not, however, have biased estimates of the association with depressive symptoms unless depressed individuals respond differently to wearing accelerometers. As this is the baseline sample of an RCT to improve CVD risk by increasing PA and reducing weight, there is the risk of a participation bias in our sample. The participants may be more interested in exercise than the general population, as they have consented in taking part in a behaviour change study. The percentage of female participants was small (14.5%), reflecting the lower QRisk2 sores in females.[24] However, data on females with high CVD risk is relevant as the

For peer review only

prevalence of depression is higher in females and they have worse outcomes following CVD events.[38]

The number of study participants with severe depressive symptoms was small, which is to be expected in a primary care population. Our analysis showed that depressive symptom scores were significantly higher in individuals who failed to provide the requested five wear days. We tried to minimise any measurement bias by depression by including all participants with as little as one valid wear day. Finally, as in any study with missing outcome data, we cannot exclude potential bias if missing values do depend on unobserved values (not missing at random).[39]

Comparison with other studies

Normative data suggests that between 2,000 and 9,000 daily steps are a typical estimate of usual daily activity levels in healthy older adults (≥50 years).[13] Our sample falls into that range with an average of 6,515 daily steps. The high inter-individual variability in step count, in our case an IQR of 3,510, is also similar to findings in previous studies.[11,12,15] Women seem to be especially likely to have a sedentary lifestyle, particularly on weekends. Ethnicity and BMI showed a significant influence on step count, as reported in previous studies.[11,13,14] The role of education level for PA continues to be uncertain with significant differences in daily steps between education levels in Model 1, but only trend level significance in Model 2. Similarly, we found that individuals with moderate alcohol consumption walked more than abstainers (significant difference in Model 1, trend level significance in Model 2), which has been reported previously in accelerometer and large population based studies.[40,41] One speculation is that these participants might be what is termed 'social drinkers' who are perhaps more likely to travel for a drink e.g. to the pub, whereas abstainers might have an advanced disease or are socially isolated. These findings suggest prevention programmes to increase PA should be targeted at specific subgroups of people at high risk of CVD, as well as the general population.

For peer review only

The prevalence of clinically relevant depressive symptoms (PHQ-9 score ≥10) in our sample was 4% which is midway between the reported UK prevalence of 2.8% in the general population (2.6% in males, 3.0% in females)[38] and 7% in CVD patients.[42] It is important to note that these statistics refer to current episodes of depressive symptoms (within the past two weeks). Lifetime prevalence of depression in the UK is reported to be higher, at around 25.8%,[43] and may also be associated with reduced PA to a greater or lesser extent, but the difference in PA levels between the two constructs of depressive symptoms have not yet been examined.

Our participants did not have CVD and were not on clinical primary care registers for other long-term conditions. However, they were at high risk of developing CVD and the prevalence rate of depressive symptoms were higher than in the general population. This is consistent with the results of a recent Danish register study which reported that depression might be an antecedent for CVD events and mortality therefore another indicator of its importance as an easily measured risk factor.[44] Previous studies on the association between objectively measured PA and depression yielded inconclusive results. They have reported either a lack of a relationship,[19,21] an inverse association,[16–18,20] or an inverse association that disappeared when controlled for confounding.[22] The studies that reported negative associations between daily step count and depressive symptoms have been conducted in relatively small (n=96 to n=207),[17–19,22] or in highly selective samples such as severe COPD.[20] Our study is the first to examine this relationship in a large sample of community-dwelling older European adults at high risk of CVD who were not known to have a chronic disease.

Implications for clinicians

Practitioners and researchers should consider assessing depressive symptoms when assessing patients' CVD risk or their barriers to uptake of lifestyle interventions. Even mild

For peer review only

symptoms of depression (PHQ-9 scores 5 to 9) were associated with a significantly reduced average daily step count of 13.3%. Similarly, it has been shown that mild symptoms of depression increase mortality risk after acute myocardial infarction.[45] A Cochrane review and a meta-meta-analysis on the effectiveness of PA interventions for the treatment of depression in clinical[46] and non-clinical samples[47] summarised that there appeared to be a moderate positive effect of increased PA on depressive symptoms. However, when stratified by quality of studies, the Cochrane review found no statistically significant evidence in methodologically robust RCTs that exercise was more effective than psychological or pharmacological therapies.[46] Nevertheless, we still found that depressive symptoms are associated with lower PA levels in individuals at high risk of CVD, which highlights the importance of screening and optimizing conventional depression management[48] to reduce depressive symptoms, which could help lower CVD risk.[3,4]

Implications for future work

The varying patterns of PA in individuals at high risk of CVD indicate that it might be most efficient to target PA interventions specifically towards those with lowest activity ratings, e.g. females, those from African, Caribbean, or Asian ethnicities, those with higher BMIs or current smokers. Additionally, our results suggest that there is a moderate association between depressive symptoms and daily step count in individuals at high risk of CVD and that this should be considered as a potential modifying factor in clinical trials for lifestyle interventions in CVD research.

For peer review only

ACKNOWLEDGEMENTS

We would like to thank the research team, healthy lifestyle facilitators, patient volunteers and the South London Clinical Research Network, participating general practices and their patients. We would also like to thank the Trial Steering Committee: Professor Steve Iliffe (Chair), University College London; Professor Tom Marshall, University of Birmingham; Professor James Carpenter, London School of Hygiene and Tropical Medicine, MRC Clinical Trials Unit; Dr Tim Anstiss, independent medical doctor and consultant. We would also like to thank the Data Ethics and Monitoring Committee: Professor Betty Kirkwood (previous Chair), London School of Hygiene and Tropical Medicine and Professor Helen Weiss (Chair), London School of Hygiene and Tropical Medicine; Professor Stephanie Taylor, Queen Mary University of London, Barts and The London School of Medicine and Dentistry, Blizard Institute; Dr David Blane, Imperial College London.

COMPETING INTERESTS STATEMENT

KI discloses that she has received honorarium from Eli-Lilly, Sanofi, Janssen and Sunovion for lectures at educational events. However, she states that this does not constitute a conflict of interest for the present study. The other authors declare no conflict of interest.

FUNDING

The MOVE IT study is funded by the National Institute for Health Research - Health Technology Assessment Programme (NIHR-HTA) (Project: 10/62/03) and work will be published in the Health Technology Assessment journal. The research attachment of VML to King's College London in London, UK, was funded by the International Network on Diabetes and Depression (IN2D), which is financed by the German Federal Ministry of Education and Research (BMBF). DS and KI received partial funding by the National Institute for Health Research (NIHR) Biomedical Research Centre at South London, and Maudsley NHS

For peer review only

Foundation Trust and King's College London. The views expressed are those of the author(s) and not necessarily those of the NHS, the NIHR or the Department of Health.

Author Contributions

KI drafted the initial hypothesis. VML developed the protocol, especially supported by AB who, as trial manager, had knowledge of all study particulars and had helped acquire the data. Further, VML wrote the first draft of the report, and performed the literature search Data analysis was conducted by DS, DGC, and VML. JLT, MA, AG and KW helped interpret the data and gave important feedback in the revision process. SRB contributed to the research funding, KI and SRB coordinated the research and oversaw the project. All authors critically reviewed the manuscript for important intellectual content and approved the final manuscript.

Ethics approval

Ethical approval was granted by the Dulwich Ethics Committee (reference: 12/LO/0917) and all participants gave written informed consent.

Data sharing statement

The full data set and statistical code are available from the corresponding author Prof. Khalida Ismail at khalida.2.ismail@kcl.ac.uk upon reasonable request.

For peer review only

REFERENCES

1 Mendis S, Puska P, Norrving B, *et al.*, editors. *Global atlas on cardiovascular disease prevention and control*. Geneva: World Health Organization in collaboration with the World Heart Federation and the World Stroke Organization 2011.

2 World Health Organization. Global Health Estimates 2015: Disease burden by Cause, Age, Sex, by Country and by Region, 2000-2015. 2016.http://www.who.int/healthinfo/global_burden_disease/estimates/en/index2.html (accessed 14 May 2017).

3 Lee I-M, Shiroma EJ, Lobelo F, *et al.* Effect of physical inactivity on major non-communicable diseases worldwide: an analysis of burden of disease and life expectancy. *The Lancet* 2012;**380**:219–29. doi:10.1016/S0140-6736(12)61031-9

4 Carney RM, Freedland KE. Depression and coronary heart disease. *Nat Rev Cardiol* 2016;**14**:145–55. doi:10.1038/nrcardio.2016.181

5 Hare DL, Toukhsati SR, Johansson P, *et al.* Depression and cardiovascular disease: a clinical review. *Eur Heart J* 2014;**35**:1365–72. doi:10.1093/eurheartj/eht462

6 Tudor-Locke C, Ainsworth BE, Whitt MC, *et al.* The relationship between pedometer-determined ambulatory activity and body composition variables. *Int J Obes Relat Metab Disord J Int Assoc Study Obes* 2001;**25**:1571–8. doi:10.1038/sj.ijo.0801783

7 Brown H, Becker F, Antwi K. Association Between Lipid Biomarkers, Physical Activity, and Socioeconomic Status in a Population-Based Cross-Sectional Study in the UK. *Sports Med - Open* 2016;**2**. doi:10.1186/s40798-016-0049-9

8 Hamer M, Chida Y. Walking and primary prevention: a meta-analysis of prospective cohort studies. *Br J Sports Med* 2008;**42**:238–43. doi:10.1136/bjsm.2007.039974

9 Prince SA, Adamo KB, Hamel M, *et al.* A comparison of direct versus self-report measures for assessing physical activity in adults: a systematic review. *Int J Behav Nutr Phys Act* 2008;**5**:56. doi:10.1186/1479-5868-5-56

10 Taraldsen K, Chastin SFM, Riphagen II, *et al.* Physical activity monitoring by use of accelerometer-based body-worn sensors in older adults: A systematic literature review of current knowledge and applications. *Maturitas* 2012;**71**:13–9. doi:10.1016/j.maturitas.2011.11.003

11 Tudor-Locke C, Ham SA, Macera CA, *et al.* Descriptive epidemiology of pedometer-determined physical activity. *Med Sci Sports Exerc* 2004;**36**:1567–73.

12 Inoue S, Ohya Y, Odagiri Y, *et al.* Sociodemographic Determinants of Pedometer-Determined Physical Activity Among Japanese Adults. *Am J Prev Med* 2011;**40**:566–71. doi:10.1016/j.amepre.2010.12.023

13 Tudor-Locke C, Hart TL, Washington TL. Expected values for pedometer-determined physical activity in older populations. *Int J Behav Nutr Phys Act* 2009;**6**:59. doi:10.1186/1479-5868-6-59

14 Davis MG, Fox KR, Hillsdon M, *et al.* Objectively Measured Physical Activity in a Diverse Sample of Older Urban UK Adults: *Med Sci Sports Exerc* 2011;**43**:647–54. doi:10.1249/MSS.0b013e3181f36196

For peer review only

15 Kari JT, Pehkonen J, Hirvensalo M, *et al.* Income and Physical Activity among Adults: Evidence from Self-Reported and Pedometer-Based Physical Activity Measurements. *PLOS ONE* 2015;**10**:e0135651. doi:10.1371/journal.pone.0135651

16 Drieling RL, Goldman Rosas L, Ma J, *et al.* Community Resource Utilization, Psychosocial Health, and Sociodemographic Factors Associated with Diet and Physical Activity among Low-Income Obese Latino Immigrants. *J Acad Nutr Diet* 2014;**114**:257–65. doi:10.1016/j.jand.2013.07.025

17 Yoshiuchi K, Nakahara R, Kumano H, *et al.* Yearlong Physical Activity and Depressive Symptoms in Older Japanese Adults: Cross-Sectional Data from the Nakanojo Study. *Am J Geriatr Psychiatry* 2006;**14**:621–4. doi:10.1097/01.JGP.0000200602.70504.9c

18 Alosco ML, Spitznagel MB, Miller L, *et al.* Depression is associated with reduced physical activity in persons with heart failure. *Health Psychol* 2012;**31**:754–62. doi:10.1037/a0028711

19 Dontje ML, van der Wal MHL, Stolk RP, *et al.* Daily Physical Activity in Stable Heart Failure Patients: *J Cardiovasc Nurs* 2013;**29**:218–26. doi:10.1097/JCN.0b013e318283ba14

20 Barriga S, Rodrigues F, Bárbara C. Factors that influence physical activity in the daily life of male patients with chronic obstructive pulmonary disease. *Rev Port Pneumol* 2014;**20**:131–7. doi:10.1016/j.rppneu.2013.09.004

21 Di Marco F, Terraneo S, Roggi MA, *et al.* Physical Activity Impairment in Depressed COPD Subjects. *Respir Care* 2014;**59**:726–34. doi:10.4187/respcare.02848

22 Harris TJ, Owen CG, Victor CR, *et al.* What factors are associated with physical activity in older people, assessed objectively by accelerometry? *Br J Sports Med* 2009;**43**:442–50. doi:10.1136/bjsm.2008.048033

23 Burton C, McKinstry B, Szentagotai Tătar A, *et al.* Activity monitoring in patients with depression: A systematic review. *J Affect Disord* 2013;**145**:21–8. doi:10.1016/j.jad.2012.07.001

24 Collins GS, Altman DG. Predicting the 10 year risk of cardiovascular disease in the United Kingdom: independent and external validation of an updated version of QRISK2. *BMJ* 2012;**344**:e4181–e4181. doi:10.1136/bmj.e4181

25 Bayley A, de Zoysa N, Cook DG, *et al.* Comparing the effectiveness of an enhanced MOtiVational intErviewing InTervention (MOVE IT) with usual care for reducing cardiovascular risk in high risk subjects: study protocol for a randomised controlled trial. *Trials* 2015;**16**. doi:10.1186/s13063-015-0593-5

26 Kroenke K, Spitzer RL, Williams JBW. The PHQ-9: Validity of a brief depression severity measure. *J Gen Intern Med* 2001;**16**:606–13. doi:10.1046/j.1525-1497.2001.016009606.x

27 Manea L, Gilbody S, McMillan D. A diagnostic meta-analysis of the Patient Health Questionnaire-9 (PHQ-9) algorithm scoring method as a screen for depression. *Gen Hosp Psychiatry* 2015;**37**:67–75. doi:10.1016/j.genhosppsych.2014.09.009

28 Lee JA, Williams SM, Brown DD, *et al.* Concurrent validation of the Actigraph gt3x+, Polar Active accelerometer, Omron HJ-720 and Yamax Digiwalker SW-701 pedometer

For peer review only

step counts in lab-based and free-living settings. *J Sports Sci* 2015;**33**:991–1000. doi:10.1080/02640414.2014.981848

29 Trost SG, Mciver KL, Pate RR. Conducting Accelerometer-Based Activity Assessments in Field-Based Research: *Med Sci Sports Exerc* 2005;**37**:S531–43. doi:10.1249/01.mss.0000185657.86065.98

30 Jané-Llopis E, Matytsina I. Mental health and alcohol, drugs and tobacco: a review of the comorbidity between mental disorders and the use of alcohol, tobacco and illicit drugs. *Drug Alcohol Rev* 2006;**25**:515–36. doi:10.1080/09595230600944461

31 Babor TF, Higgins-Biddle JC, Saunders JB, *et al. AUDIT The Alcohol Use Disorders Identification Test. Guidelines for Use in Primary Care.* Second Edition. World Health Organization Department of Mental Health and Substance Dependence 2001.

32 Actigraph Software Department. *ActiLife 6 User's Manual*. Pensacola, FL: 2012.

33 Rothman KJ. No adjustments are needed for multiple comparisons. *Epidemiol Camb Mass* 1990;**1**:43–6.

34 Shek DTL, Ma CMS. Longitudinal Data Analyses Using Linear Mixed Models in SPSS: Concepts, Procedures and Illustrations. *Sci World J* 2011;**11**:42–76. doi:10.1100/tsw.2011.2

35 Brown H, Prescott R. *Applied mixed models in medicine*. Third edition. Chichester, West Sussex ; Hoboken: John Wiley & Sons Inc 2015.

36 Lang TA, Secic M. *How to report statistics in medicine: annotated guidelines for authors, editors, and reviewers*. 2nd Edition. ACP Press 2006.

37 Hsueh Y. The Hawthorne experiments and the introduction of Jean Piaget in American industrial psychology, 1929-1932. *Hist Psychol* 2002;**5**:163–89. doi:10.1037//1093-4510.5.2.163

38 Paykel ES, Brugha T, Fryers T. Size and burden of depressive disorders in Europe. *Eur Neuropsychopharmacol* 2005;**15**:411–23. doi:10.1016/j.euroneuro.2005.04.008

39 Rubin DB. Inference and missing data. *Biometrika* 1976;**63**:581–92. doi:10.1093/biomet/63.3.581

40 French MT, Popovici I, Maclean JC. Do Alcohol Consumers Exercise More? Findings from a National Survey. *Am J Health Promot* 2009;**24**:2–10. doi:10.4278/ajhp.0801104

41 Westerterp KR, Meijer EP, Goris AHC, *et al.* Alcohol energy intake and habitual physical activity in older adults. *Br J Nutr* 2004;**91**:149. doi:10.1079/BJN20031013

42 Walters P, Barley EA, Mann A, *et al.* Depression in Primary Care Patients with Coronary Heart Disease: Baseline Findings from the UPBEAT UK Study. *PLoS ONE* 2014;**9**:e98342. doi:10.1371/journal.pone.0098342

43 Smith DJ, Nicholl BI, Cullen B, *et al.* Prevalence and Characteristics of Probable Major Depression and Bipolar Disorder within UK Biobank: Cross-Sectional Study of 172,751 Participants. *PLoS ONE* 2013;**8**:e75362. doi:10.1371/journal.pone.0075362

For peer review only

44 Sundboll J, Schmidt M, Adelborg K, *et al.* Impact of pre-admission depression on mortality following myocardial infarction. *Br J Psychiatry* 2017;**210**:356–61. doi:10.1192/bjp.bp.116.194605

45 Bush DE, Ziegelstein RC, Tayback M, *et al.* Even minimal symptoms of depression increase mortality risk after acute myocardial infarction. *Am J Cardiol* 2001;**88**:337–41. doi:10.1016/S0002-9149(01)01675-7

46 Cooney GM, Dwan K, Greig CA, *et al.* Exercise for depression. *Cochrane Database Syst Rev* 2013. doi:10.1002/14651858.cd004366.pub6

47 Rebar AL, Stanton R, Geard D, *et al.* A meta-meta-analysis of the effect of physical activity on depression and anxiety in non-clinical adult populations. *Health Psychol Rev* 2015;**9**:366–78. doi:10.1080/17437199.2015.1022901

48 National Institute for Clinical Excellence. Depression: the treatment and management of depression in adults (update). *Clinical guidelines, CG90* 2009.

Figure 1: The association between depressive symptoms and daily step count at baseline in adults with high cardiovascular risk in a randomised controlled trial assessing the effectiveness of an intensive lifestyle intervention to improve physical activity and reduce weight.

For peer review only

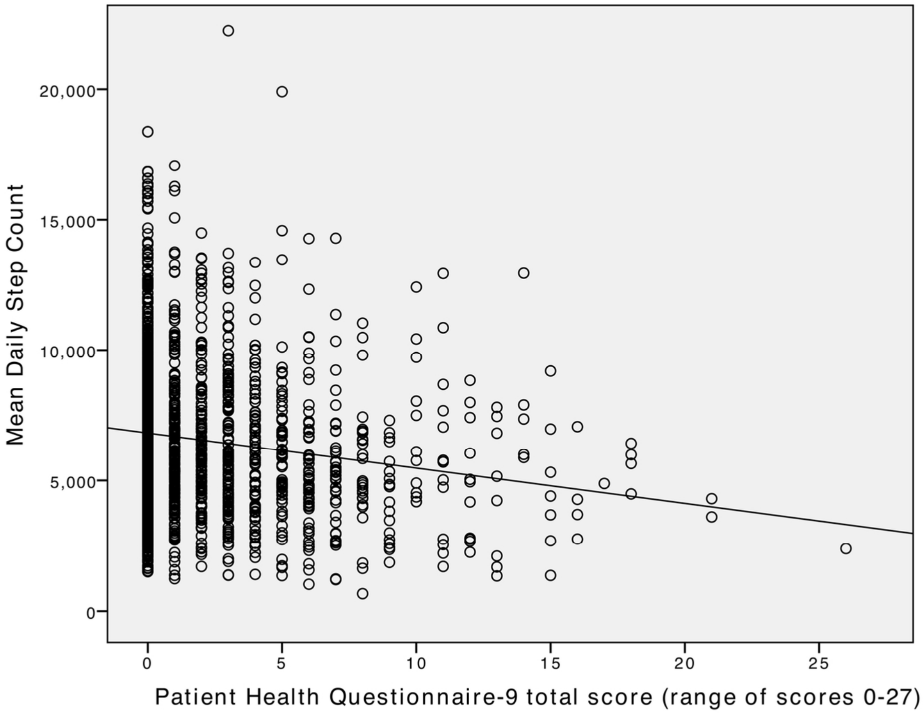

Figure 1: The association between depressive symptoms and daily step count at baseline in adults with high cardiovascular risk in a randomised controlled trial assessing the effectiveness of an intensive lifestyle intervention to improve physical activity and reduce weight.

127x96mm (300 x 300 DPI)

Supplementary Table 1: Estimated effects (coefficients and relative % change) of individual characteristics on average daily steps in subjects at high risk of CVD with ≥5 valid wear days

Variables	Model 1[a]		Model 2[b]	
	Coefficient	Relative % Change (95% CI)	Coefficient	Relative % Change (95% CI)
**Constant**	8.834		8.942	
**Basic confounders**				
**Age** (impact of additional year)	0.002	0.2 (-0.3 to 0.7)	-0.013***	-1.3 (-1.8 to -0.7)
**Gender** Male	0	0	0	0
Female	-0.239***	-21.3 (-25.9 to -16.4)	-0.143***	-13.4 (-18.4 to -7.9)
**Day** Weekday	0	0	0	0
Weekend	-0.162***	-14.9 (-16.6 to -13.2)	-0.161***	-14.9 (-16.6 to -13.2)
**Season** Spring	0	0	0	0
Summer	-0.001	-0.1 (-6.1 to 6.3)	0.011	1.1 (-4.8 to 7.3)
Autumn	-0.005	-0.5 (-6.4 to 5.8)	-0.005	-0.5 (-6.2 to 5.6)
Winter	-0.095**	-9.0 (-14.4 to -3.3)	-0.102**	-9.7 (-14.9 to -4.2)
**a) Depressive Symptoms constant**	8.854			
None (PHQ-9: 0 - 4)	0	0	0	0
Mild (PHQ-9: 5 - 9)	-0.149***	-13.9 (-19.4 to -7.9)	-0.124***	-11.7 (-17.2 to -5.8)
Moderate/Severe (PHQ-9≥10)	-0.171**	-15.7 (-24.7 to -5.6)	-0.135**	-12.6 (-21.5 to -2.7)
**b) Ethnicity constant**	8.848			
White	0	0	0	0
Black/Asian/Other	-0.136***	-12.7 (-18.5 to -6.4)	-0.193***	-17.5 (-23.1 to -11.5)
**c) Education level constant**	8.872			
Level 3: A Level or higher	0	0	0	0
Level 2: O Level/GCSE/NVQ	-0.072**	-7.0 (-11.5 to -2.1)	-0.058*	-5.6 (-10.1 to -1.0)
Level 1: no formal qualification	-0.087**	-8.3 (-13.0 to -3.4)	-0.075**	-7.2 (-11.8 to -2.5)
**d) BMI constant**	8.830			
Impact of one unit BMI increase	-0.025***	-2.5 (-3.0 to -2.0)	-0.028***	-2.8 (-3.3 to -2.3)
**e) Smoking status constant**	8.863			
Never smoked	0	0	0	0
Ex-smoker	-0.016	-1.5 (-6.1 to 3.3)	-0.027	-2.7 (-7.1 to 2.0)
Current smoker	-0.120**	-11.3 (-17.2 to -5.0)	-0.189***	-17.2 (-22.6 to -11.4)
**f) AUDIT score constant**	8.844			
Low risk (score 1-7)	0	0	0	0
Possibly harmful (score ≥8)	0.004	0.4 (-5.3 to - 6.5)	0.035	3.6 (-2.1 to 9.7)
Abstainer (score 0)	-0.119**	-11.3 (-17.3 to -4.8)	-0.089*	-8.6 (-14.7 to -1.9)

[a]*Model 1: all variables adjusted for basic confounders (age, gender, day, season). Note: There is a constant for the basic confounders only model and separate constants for this model adjusted for each additional confounder (a-f).* [b]*Model 2: all variables mutually adjusted, thus there is only one constant for the whole model. Reference group within each category is set to 0. AUDIT:* Alcohol Use Disorders Identification Test. *Sign. levels: *<.05, **<.01, ***<.001.*

For peer review only

Supplementary Table 2: Estimated effects (coefficients and relative % change) of individual characteristics on average daily steps in subjects at high risk of CVD with a QRisk2 ≥ 20%

Variables		Model 1[a]		Model 2[b]	
		Coefficient	Relative % Change (95% CI)	Coefficient	Relative % Change (95% CI)
**Constant**		8.803		8.918	
**Basic confounders**					
**Age** (impact additional year)		0.004***	0.4 (-0.1 to 1.0)	-0.011***	-1.1 (-1.7 to -0.5)
**Gender**	Male	0	0	0	0
	Female	-0.225***	-20.1 (-25.2 to -14.7)	-0.133***	-12.4 (-18.0 to -6.5)
**Day**	Weekday	0	0	0	0
	Weekend	-0.162***	-15.0 (-16.7 to -13.2)	-0.162***	-15.0 (-16.7 to -13.1)
**Season**	Spring	0	0	0	0
	Summer	-0.002	-0.3 (-6.4 to 6.4)	0.0128	1.3 (-4.8 to 7.8)
	Autumn	-0.015	-1.5 (-7.6 to 5.0)	-0.018	-1.8 (-7.6 to 4.5)
	Winter	-0.094**	-9.0 (-14.6 to -3.0)	-0.103**	-9.8 (-15.2 to -4.0)
**a) Depressive Symptoms constant**		8.830			
None (PHQ-9: 0 - 4)		0	0	0	0
Mild (PHQ-9: 5 - 9)		-0.177***	-16.2 (-21.7 to -10.4)	-0.148***	-13.8 (-19.3 to -7.9)
Moderate/Severe (PHQ-9≥10)		-0.234***	-20.9 (-29.3 to -11.5)	-0.206***	-18.6 (-26.9 to -9.3)
**b) Ethnicity constant**		8.817			
White		0	0	0	0
Black/Asian/Other		-0.133***	-12.4 (-18.6 to -5.7)	-0.181***	-16.6 (-22.6 to -10.1)
**c) Education level constant**		8.835			
Level 3: A Level or higher		0	0	0	0
Level 2: O Level/GCSE/NVQ		-0.059*	-5.7 (-10.6 to -0.6)	-0.047	-4.6 (-9.4 to -0.4)
Level 1: no formal qualification		-0.073**	-7.0 (-11.9 to -1.9)	-0.056*	-5.5 (-10.3 to -0.4)
**d) BMI constant**		8.804			
BMI per one unit increase		-0.023***	-2.3 (-2.8 to -1.8)	-0.026***	-2.6 (-3.1 to -2.1)
**e) Smoking status constant**		8.835			
Never smoked		0	0	0	0
Ex-smoker		-0.015	-1.5 (-6.4 to 3.7)	-0.028	-2.8 (-7.5 to 2.2)
Current smoker		-0.139***	-12.9 (-19.0 to -6.5)	-0.203***	-18.4 (-24.0 to -12.3)
**f) AUDIT score constant**		8.810			
Low risk (score 1-7)		0	0	0	0
Possibly harmful (score ≥8)		0.011	1.1 (-4.9 to 7.5)	0.042	4.25 (-1.7 to 10.7)
Abstainer (score 0)		-0.095*	-9.1 (-15.6 to -2.1)	-0.072	-7.0 (-13.6 to 0.2)

[a]*Model 1: all variables adjusted for basic confounders (age, gender, day, season). Note: There is a constant for the basic confounders only model and separate constants for this model adjusted for each additional confounder (a-f).* [b]*Model 2: all variables mutually adjusted, thus there is only one constant for the whole model. Reference group within each category is set to 0. AUDIT:* Alcohol Use Disorders Identification Test. *Sign. levels: *<.05, **<.01, ***<.001.*

For peer review only

STROBE 2007 (v4) Statement—Checklist of items that should be included in reports of *cross-sectional studies*

**Section/Topic**	**Item #**	**Recommendation**	**Reported on page #**
**Title and abstract**	1	(*a*) Indicate the study's design with a commonly used term in the title or the abstract	1
		(*b*) Provide in the abstract an informative and balanced summary of what was done and what was found	2
**Introduction**			
Background/rationale	2	Explain the scientific background and rationale for the investigation being reported	3-5
Objectives	3	State specific objectives, including any prespecified hypotheses	4
**Methods**			
Study design	4	Present key elements of study design early in the paper	5-7
Setting	5	Describe the setting, locations, and relevant dates, including periods of recruitment, exposure, follow-up, and data collection	5
Participants	6	(*a*) Give the eligibility criteria, and the sources and methods of selection of participants	5
Variables	7	Clearly define all outcomes, exposures, predictors, potential confounders, and effect modifiers. Give diagnostic criteria, if applicable	5-7
Data sources/ measurement	8*	For each variable of interest, give sources of data and details of methods of assessment (measurement). Describe comparability of assessment methods if there is more than one group	5-7
Bias	9	Describe any efforts to address potential sources of bias	7-9
Study size	10	Explain how the study size was arrived at	9
Quantitative variables	11	Explain how quantitative variables were handled in the analyses. If applicable, describe which groupings were chosen and why	5-7
Statistical methods	12	(*a*) Describe all statistical methods, including those used to control for confounding	7-9
		(*b*) Describe any methods used to examine subgroups and interactions	-
		(*c*) Explain how missing data were addressed	7
		(*d*) If applicable, describe analytical methods taking account of sampling strategy	-
		(*e*) Describe any sensitivity analyses	9

For peer review only

**Results**			
Participants	13*	(a) Report numbers of individuals at each stage of study—eg numbers potentially eligible, examined for eligibility, confirmed eligible, included in the study, completing follow-up, and analysed	9-10
		(b) Give reasons for non-participation at each stage	9-10
		(c) Consider use of a flow diagram	-
Descriptive data	14*	(a) Give characteristics of study participants (eg demographic, clinical, social) and information on exposures and potential confounders	9-11
		(b) Indicate number of participants with missing data for each variable of interest	9-11
Outcome data	15*	Report numbers of outcome events or summary measures	9-10
Main results	16	(*a*) Give unadjusted estimates and, if applicable, confounder-adjusted estimates and their precision (eg, 95% confidence interval). Make clear which confounders were adjusted for and why they were included	12-13
		(*b*) Report category boundaries when continuous variables were categorized	6-9
		(*c*) If relevant, consider translating estimates of relative risk into absolute risk for a meaningful time period	-
Other analyses	17	Report other analyses done—eg analyses of subgroups and interactions, and sensitivity analyses	8-9
**Discussion**			
Key results	18	Summarise key results with reference to study objectives	14
Limitations	19	Discuss limitations of the study, taking into account sources of potential bias or imprecision. Discuss both direction and magnitude of any potential bias	14-15
Interpretation	20	Give a cautious overall interpretation of results considering objectives, limitations, multiplicity of analyses, results from similar studies, and other relevant evidence	15-16
Generalisability	21	Discuss the generalisability (external validity) of the study results	16-17
**Other information**			
Funding	22	Give the source of funding and the role of the funders for the present study and, if applicable, for the original study on which the present article is based	18

*Give information separately for cases and controls in case-control studies and, if applicable, for exposed and unexposed groups in cohort and cross-sectional studies.

Note: An Explanation and Elaboration article discusses each checklist item and gives methodological background and published examples of transparent reporting. The STROBE checklist is best used in conjunction with this article (freely available on the Web sites of PLoS Medicine at http://www.plosmedicine.org/, Annals of Internal Medicine at http://www.annals.org/, and Epidemiology at http://www.epidem.com/). Information on the STROBE Initiative is available at www.strobe-statement.org.

For peer review only
